# Weather field reconstruction using aircraft surveillance data and a novel meteo-particle model

**DOI:** 10.1371/journal.pone.0205029

**Published:** 2018-10-03

**Authors:** Junzi Sun, Huy Vû, Joost Ellerbroek, Jacco M. Hoekstra

**Affiliations:** Control and Simulation, Faculty of Aerospace Engineering, Delft University of Technology, Kluyverweg 1, 2629 HS Delft, the Netherlands; Universidade de Vigo, SPAIN

## Abstract

Wind and temperature data are important parameters in aircraft performance studies. The lack of accurate measurements of these parameters forces researchers to rely on numerical weather prediction models, which are often filtered for a larger area with decreased local accuracy. Aircraft, however, also transmit information related to weather conditions, in response to interrogation by air traffic controller surveillance radars. Although not intended for this purpose, aircraft surveillance data contains information that can be used for weather models. This paper presents a method that can be used to reconstruct a weather field from surveillance data that can be received with a simple 1090 MHz receiver. Throughout the paper, we answer two main research questions: how to accurately infer wind and temperature from aircraft surveillance data, and how to reconstruct a real-time weather grid efficiently. We consider aircraft as moving sensors that measure wind and temperature conditions indirectly at different locations and flight levels. To address the first question, aircraft barometric altitude, ground velocity, and airspeed are decoded from down-linked surveillance data. Then, temperature and wind observations are computed based on aeronautical speed conversion equations. To address the second question, we propose a novel Meteo-Particle (MP) model for constructing the wind and temperature fields. Short-term local prediction is also possible by employing a predictor layer. Using an unseen observation test dataset, we are able to validate that the mean absolute errors of inferred wind and temperature using MP model are 67% and 26% less than using the interpolated model based on GFS reanalysis data.

## 1 Introduction

Using aircraft as weather sensors is a recent development in air traffic management and meteorological research. Traditionally, aircraft obtain weather updates from Air Traffic Services, which they use to optimize their trajectory and speed, to best adapt to wind conditions and to avoid areas of extreme conditions. These meteorological updates come mostly from ground-based observations, such as radar surveillance or weather observation stations, or from forecast systems [1] [2]. In addition to this, local meteorological conditions are also computed by the aircraft, using observations from on-board air data sensors. Existing technologies such as Aircraft Meteorological Data Relay (AMDAR) [3] and Meteorological Routine Air Report (MRAR) allow aircraft to down-link these meteorological data either through the Aircraft Communications Addressing and Reporting System (ACARS) or using a technology called Selective Interrogation (Mode-S).

Both AMDAR and MRAR are unencrypted broadcast data, which means that anyone can set up receivers to intercept these data. However, as part of ACARS, the legality of intercepting AMDAR is questionable in certain countries. As for MRAR, the number of aircraft that broadcast this information is limited, since most aircraft choose not to enable the MRAR capability. In addition, this information is not always interrogated by Air Traffic Control surveillance radars.

Besides explicitly transmitted meteorological data, aircraft state information can also be used to infer local meteorological conditions. The state information is traditionally acquired using the primary surveillance radar for aircraft position, complemented by the secondary radar, which interrogates aircraft to obtain on-board data on, e.g., airspeed. Nowadays, aircraft are also equipped with automatic broadcast technologies, which ground reception of (unencrypted) state information without the need for interrogation. The details of these technologies are explained later in this paper. Several studies have proposed to use such flight data to estimate wind conditions at the location of an aircraft. We can categorize these studies into three categories:

- *Estimation of wind from ground-based trajectory observations*: This concept assumes a quasi-constant wind velocity and aircraft airspeed during turning maneuvers. Under these assumptions, the wind velocity vector can be estimated dynamically using observations of aircraft ground speed in combination with Bayesian filtering. A method using radar track data, based on this concept, was first proposed in 1989 [4]. Later on, variations and extensions of the method were implemented [5] [6] [7]. Nowadays, with Automatic Dependent Surveillance-Broadcast (ADS-B) transponders installed on most commercial aircraft, simple, ground-based monitoring of aircraft states through ADS-B has become a possibility, and the use of ADS-B data for weather determination is being explored [8] [9].
- *Estimation of aircraft local weather conditions from interrogated aircraft data*: To provide more aircraft state information to air traffic controllers, Mode-S secondary radar surveillance was developed as a complementary source of information to radar. Mode-S is designed to interrogate specific aircraft states individually, such as airspeed, intentions, and turn performance. A series of studies conducted by the Dutch Meteorological Institute presented wind data constructed from Mode-S and MRAR [10] [11]. Other research combined MRAR data and Kalman filtering to construct weather conditions [12]. In addition to the direct wind information in MRAR (which is rarely requested), the airspeed of aircraft is down-linked upon interrogation using Mode-S. This information can be used to compute wind as the difference between aircraft airspeed and ground speed [13]. Temperature, on the other hand, can be derived from ADS-B data alone, based on the difference between Global Navigation Satellite System (GNSS) height and barometric altitude, which are both broadcast with ADS-B [14].
- *Wind field estimation based on multiple wind measurements*: While most of the above studies focused on deriving the local meteorological conditions of an aircraft, other studies tried to extend such methods to wind field or multiple aircraft scenarios. For example, a Hidden Markov model was used to update a wind grid, based on measurements from multiple aircraft by Hollister [4]. Delahaye and De Leeghe used non-linear Kalman filters on radar and ADS-B data, respectively [6] [8]. The least-squares method was also employed to construct wind fields from multiple aircraft measurements [15]. Finally, a concept using machine learning based on Gaussian Process was also proposed [16].

The methods described above reveal the potential of using aircraft surveillance data, but they are not without limitations. For example, some studies considered only the wind, while other studies were based on data from air traffic controllers. Rarely has the potential of large quantities of streaming aircraft surveillance data been exploited to its full potential. Moreover, it is hard to identify a method that is fast to compute and easy reproducible.

In this paper, we focus on two main research questions: 1) how to accurately infer both wind and temperature measurements from Mode-S and ADS-B data; 2) how to reconstruct the real-time weather grid efficiently. In addition to answering these two main questions, we also construct an open-source model that can be re-used for future research.

Weather data assimilation is a well-established area of research. Existing methods include Kalman filtering [17] and 3D/4D variational assimilation [18] [19]. Unlike traditional weather observations such as those from ground stations and weather balloons, there is an abundant amount of observation data being generated from aircraft flight. In our proposed model, temperature and wind are first computed based on the ADS-B and Mode-S down-link messages. Within the 400 kilometers radius of a typical ground receiver setup, it is common to obtain more than 50 weather observations per second with normal operational air traffic density. The characteristics of weather observations from aircraft are:

- Aircraft are moving objects. The measurements derived from air traffic data vary both in time and position.
- Aircraft often fly along predefined routes. As such, measurements are concentrated along a limited number of flight paths. This creates a highly uneven distribution of measurements in space.
- The interval between successive measurements is small (in the order of a single second).
- There are errors in individual wind calculations caused by different sources, such as measurement error (aircraft), transmission error (data link), and identification error (decoder).

Considering these characteristics of the data source, in this paper, we propose a model named Meteo-Particle (MP) model that is able to construct accurate local wind and temperature fields. The model is aimed at providing estimation within aviation airspace with a confidence indicator using only aircraft surveillance data. The fundamental idea of the model is to use a stochastic process to extend weather information (modeled as particles) from high-density flight paths to areas without aircraft observations. The system works on a short timescale, which can be from several minutes to an hour. It is constructed and maintained with a probabilistic nature incorporating both historical and recent weather information. The model is first validated with weather data from numerical forecast models. The variance and stability of the MP model are tested, and the error tolerance is also examined.

Before introducing the details of the MP model, it needs to be distinguished two existing concepts use the terminology: particles. These concepts are particle filtering [20] [21] and Lagrangian transportation modeling [22]. The particle filter, also called Sequential Monte Carlo, is a system state estimator, and particles are numerical approximations of probability density functions of the states. The Lagrangian transportation model is commonly used to simulate atmospheric chemistry, where the particles propagation follows atmospheric dynamics, such as fluid and heat. In the proposed MP model, the particles can be considered as the information medium, which propagates the wind and temperature measurements to surrounding areas. The propagation of MP particles is a stochastic random walk process.

The remainder of the paper is structured as follows. Section two describes the process of obtaining wind observations. Section three focuses on the Meteo-Particle model with examples. Section four discusses the ability to make short-term predictions based on the model. Section five and six detail experiments and validations, and provides an analysis of the MP model. The discussion and conclusions are presented in sections seven and eight.

## 2 Meteorological observations from ADS-B and Mode-S data

The Automatic Dependent Surveillance-Broadcast (ADS-B) is an aircraft surveillance technology that enables aircraft to automatically broadcast flight states such as location, altitude, and ground speed. In contrast to conventional surveillance technologies, ADS-B enables information exchange without the need for interrogation from air traffic controllers. Information is broadcast approximately every half of a second, providing a fast update rate of the aircraft states. A downside from the perspective of weather estimation, however, is that within ADS-B messages, only ground speed is transmitted. For the computation of wind, we need also need to obtain the airspeed of the aircraft. Here, ground speed refers to the relative speed between the aircraft and the earth, while the airspeed refers to the relative speed between the aircraft and the air. Without wind these two speeds are equal. When wind is present, it can be obtained from the difference between ground speed and airspeed vectors.

The airspeed can be acquired from the Comm-B response messages that are generated by selective interrogations (Mode-S) from the secondary surveillance radar. However, unlike ADS-B, Mode-S Comm-B replies do not contain any information on their message types. This is because only the interrogating radar knows the target aircraft and what to expect in the down-link messages. This lack of transparency in the Mode-S design poses the biggest challenge in making use of this type of open data.

### 2.1 Processing Mode-S data

Through Mode-S, different aircraft state information is down-linked to ground receivers. This information includes parameters such as aircraft position, velocity, operational parameters, and meteorological data. The Mode-S transponder maintains 255 different 56-bit wide Binary Data Store registers (BDS) that can be interrogated by ATC. These registers are indicated by two-digit hexadecimal numbers that can be requested via 25 different Down-link Formats (DF). Information in these registers is updated with a minimum interval specified by the International Civil Aviation Organization (ICAO).

Among these down-link formats, ADS-B is transmitted via DF 17 (also known as Mode-S extended squitter), while Mode-S Comm-B replies are transmitted via DF 20 and DF 21. The decoding of ADS-B messages is well documented. The interpretation of Comm-B replies is much more difficult. The challenges include determining the originating aircraft (its ICAO address), the content of the message (from BDS code), and the quality of the content (certainty of the values).

Aircraft ICAO addresses can be determined by performing a reverse parity check of the Comm-B messages. Correct ICAO addresses can only be obtained when a signal is not corrupt. If a message is corrupt (e.g. one or more bits are flipped), this will result in an incorrect ICAO address. However, error messages can be discovered by cross-referencing resulting ICAO addresses with ADS-B, which are always correct if the messages are not corrupt.

The BDS code is determined by checking several status bits and evaluating possible values contained in the messages. A status bit indicates whether its related register field (aircraft parameter) is available in the message. The implemented steps are the following: When a status bit is set to zero, all related content bits should be zero as well. Messages with different BDS codes usually have different signification status bits. Thus, multiple checks assuming different message types need to be performed to evaluate all possible types or a combination of types. It may occur that a message matches multiple BDS codes. In this paper, only uniquely identified messages are kept and used for the proposed MP model.

The quality of the Mode-S measurements is also an important factor. Values decoded from corrected messages may be incorrect due to aircraft measurements or transmission errors. The truncation of values in messages can also lower the resolution of the measurements [10]. The proposed model needs to cope with this uncertainty, in addition to the errors that are raised from incorrect BDS register identification.

Prior to this research, we designed the open-source library pyModeS [23] for decoding Mode-S Comm-B replies, which is used in this paper to handle surveillance messages. The pyModeS implements all the decoding of ADS-B data (DF 17), as well as the inference of Comm-B replies (DF 20/21).

### 2.2 Accurate models of temperature and wind speed

With ADS-B messages and Mode-S Comm-B replies, one can derive multiple features of any aircraft state. This information can be used to compute the meteorological conditions (temperature and wind) indirectly but accurately. The necessary aircraft states are:

- **Aircraft barometric altitude** (*H*_*p*_): broadcast through ADS-B (Type code: 9 to 18), high update rate, high certainty.
- **Aircraft ground speed** (*V*_gs_): broadcast through ADS-B (Type code: 19), high update rate, high certainty.
- **Aircraft true airspeed** (*V*_tas_): transmitted in Mode-S Comm-B BDS 5,0 message, low update rate, lower certainty than *V*_gs_.
- **Aircraft indicated airspeed** (*V*_ias_): transmitted in Mode-S Comm-B BDS 6,0 message, medium update rate, lower certainty than *V*_gs_.
- **Aircraft Mach number** (*M*): transmitted in Mode-S Comm-B BDS 6,0 message, medium update rate, lower certainty than *V*_gs_.

**Fig 1:**
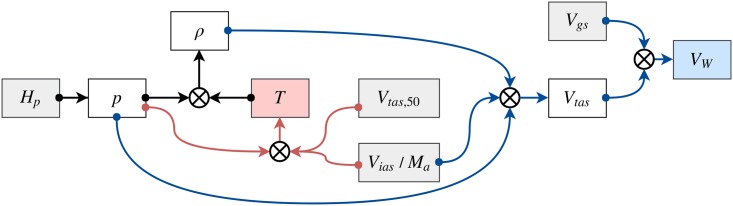
Inference of related meteorological states.

Denoting the *p*, *ρ*, *T*, *V*_*w*_ as the atmospheric pressure, air density, air temperature, and wind speed, the inference procedure is shown in the flow diagram of Fig 1.

The gray blocks represent the observable aircraft states, and the white blocks are intermediate atmospheric or aircraft states. The red line indicates the steps required to compute the temperature, while the blue lines refer to the inference of wind.

Since the update rate of actual true airspeed from BDS 5,0 messages is low, true air speed converted from indicated airspeed in BDS 6,0 messages is also used. However, such a conversion requires knowledge of the air temperature, which can be computed using true airspeed from BDS 5,0 messages in combination with the air pressure. The constants listed in Table 1 are used to compute the temperature in Eq 1:

$$\begin{array}{c} \begin{array}{cc} p & =p_{0}\cdot \text{exp}(-\frac{g_{0}}{R\;T_{0}}h)\\ T & =\frac{V_{\text{tas},50}^{2}\cdot p}{V_{\text{ias}}^{2}\cdot \rho_{0}\cdot R}\;\;M<0.3\\ T & =\frac{V_{\text{tas},50}^{2}\cdot T_{0}}{M^{2}\cdot a_{0}^{2}}\;\;M⩾0.3 \end{array} \end{array}$$ (1)

where *V*_tas,50_ is the true airspeed from the BDS 5,0 message. Due to the compressibility of air, when an aircraft flies at high speed (*M* ≥ 0.3), the Mach number has to be used instead of the indicated airspeed. Once the temperature has been derived, the true airspeed can be computed similarly:

$$\begin{array}{c} \begin{array}{cc} \rho & =\frac{p}{R\;T}\\ V_{\text{tas}} & =V_{\text{ias}}\sqrt{\frac{\rho_{0}}{\rho }}\;\;M<0.3\\ V_{\text{tas}} & =Ma_{0}\sqrt{\frac{T}{T_{0}}}\;\;M⩾0.3 \end{array} \end{array}$$ (2)

**Table 1:** Constants of International Standard Atmosphere model.

Parameter	Value	Unit	Description
*p*_0_	101325	Pa	air pressure at sea level
*ρ*_0_	1.225	kg/*m*^3^	air density at sea level
*a*_0_	340.29	m/s	speed of sound at sea level
*T*_0_	288.15	K	temperature at sea level
*R*	287.05	J/(kg ⋅ K)	gas constant at sea level

**Fig 2:**
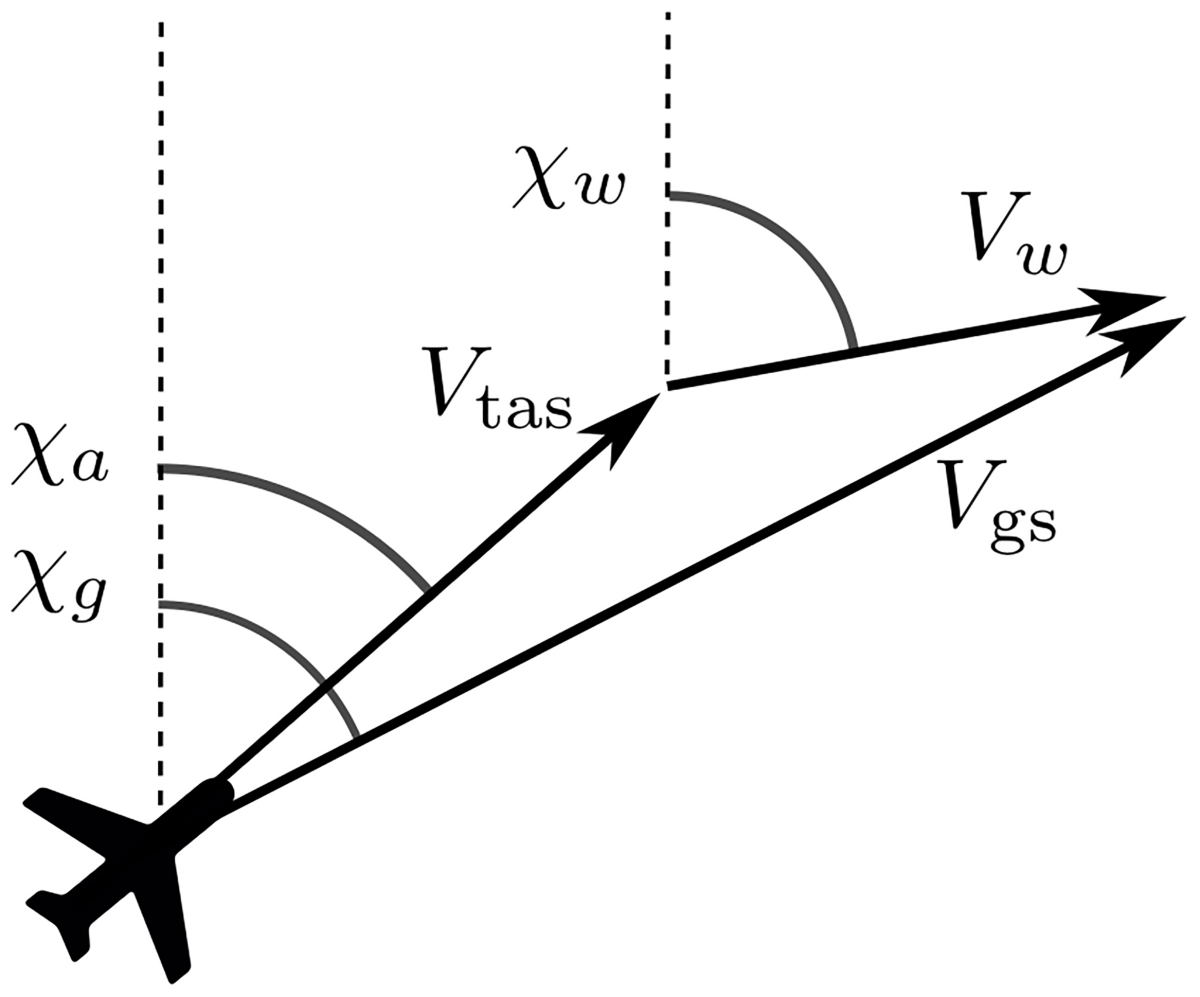
Relation between true airspeed, ground speed, and wind vector.

After the true airspeed is obtained, together with the heading, ground speed, and track angle, the wind vector is derived. Fig 2 shows the relationship between true airspeed, ground speed, and wind.

*χ*_*g*_, *χ*_*a*_, and *χ*_*w*_ are the track angle, aircraft heading, and wind direction with respect to the true north respectively. The ground speed vector is then computed according to Eq 3.

$$\begin{array}{c} {\vec{V}}_{w}={\vec{V}}_{\text{gs}}-{\vec{V}}_{\text{tas}} \end{array}$$ (3)

where the wind vector is the subtraction of true airspeed from ground speed.

## 3 The Meteo-Particle model

**Fig 3:**
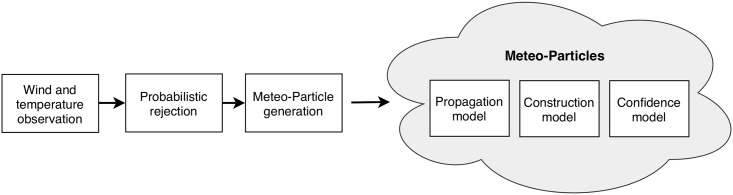
Steps and components of the MP model.

In the proposed Meteo-Particle (MP) model, the particles are modeled to represent the states of wind and temperature measurement. Particles are first generated when a new observation of wind and temperature is obtained. They propagate and decay over time according to separate models. Wind fields are constructed by combining the weighted states of all neighboring particles. The propagation of particles allows for wind to be computed at areas with low measurement density. The following section will be dedicated to a more detailed explanation of the model, methods, and exponential functions used to compute wind field and confidences levels. In Fig 3, the general steps are illustrated.

### 3.1 Assumptions

The model’s functioning depends on the following assumptions:

1. *The true states of wind and temperature are geographically stable at the level of tens of kilometers.* This assumption ensures that the atmospheric states at any location can be represented by observations made in adjacent areas. Turbulence breaks this assumption, and thus cannot be represented accurately in the model.
2. *The true states of wind and temperature of a given location are stable at the level of minutes.* This assumption ensures that the dynamics of field states do not change too rapidly. This is usually true because aircraft avoid extreme atmospheric conditions as much as possible. Nevertheless, the MP model is able to track rapid local changes by reducing the aging parameter, but at the cost of stability for the larger airspace.
3. *The burst error rate of observations from a single aircraft is reasonable not too high.* The burst error is a continuous sequence of wrong measurements from incorrect aircraft states. This error is hard to eliminate due to the uncertainty in Mode-S Comm-B reply decoding. However, with the probabilistic measurement rejection of the MP model, we can reduce the effect of burst errors.

### 3.2 Measurements and probabilistic rejection

Measurements are done at aircraft position (*x*, *y*, *z*), which is converted from the longitude, latitude, and altitude reported by the aircraft. A wind measurement is a vector represented by a west-east component (U component) *u* and a south-north component (V component) *v* at a specific location. The temperature is a scalar denoted as *τ*. The measurement array consists of all wind measurements from different aircraft at the time interval of one second in the observed airspace. It is denoted as [***x***, ***y***, ***z***, ***u***, ***v***, ***τ***].

When wind and temperature samples are derived using ADS-B and Mode-S Comm-B reply, there is a chance that the wind measurements are incorrect. This often occurs due to incorrect decoding. Although the chance of incorrect data is low, such wrong information can cause sudden variations in instantaneous wind fields. To solve this problem, a probabilistic rejection mechanism is applied.

For each new measurement ***x***: (*u*, *v*, *τ*), a probability function is constructed based on the current field. First, the mean and variance of wind and temperature states from existing particles from the same vertical level (+/- 500 meters) are computed. These are denoted as $\left(\mu_{u},\sigma_{u}^{2}\right)$ for the u component of the wind, $\left(\mu_{v},\sigma_{v}^{2}\right)$ for the v component of the wind, and $\left(\mu_{\tau },\sigma_{\tau }^{2}\right)$ for the temperature. The probability function is expressed as:

$$\begin{array}{c} \begin{array}{cc} p & =\text{exp}[-\frac{1}{2}{\left(x-\mu \right)}^{T}{\left(k_{1}\Sigma \right)}^{-1}\left(x-\mu \right)]\\ \mu & =(\mu_{u},\mu_{v},\mu_{\tau })\\ \Sigma & =[\begin{array}{c} \sigma_{u}^{2},\;0,\;0\\ 0,\;\sigma_{v}^{2},\;0\\ 0,\;0,\;\;\sigma_{\tau }^{2} \end{array}] \end{array} \end{array}$$ (4)

Hence, any new sample will be accepted with a probability of *p*. This extra step ensures a low probability of acceptance for extreme wind samples. On the other hand, due to its probabilistic nature, it will also decrease the number of correct samples that are accepted. As a trade-off, one can increase the parameter *k*_1_ for a higher tolerance. We propose to choose *k*_1_ as a value between 2 to 4.

### 3.3 Particles

A particle is defined as a point object that carries the state of the wind and temperature. Particle states consist of position (*x*_*p*_, *y*_*p*_, *z*_*p*_), origin (*x*_0_, *y*_0_, *z*_0_), representing horizontal wind components (*u*_*p*_, *v*_*p*_), temperature, *τ*_*p*_, and age (*α*).

Particles are generated when new wind measurements are observed (computed). For each new measurement [*x*, *y*, *z*, *u*, *v*, *τ*], *N* number of particles are created at the location of the aircraft.

$$\begin{array}{c} {}[\begin{array}{c} x_{p,i}\\ y_{p,i}\\ z_{p,i} \end{array}]=[\begin{array}{c} x\\ y\\ z \end{array}],\;i=1,2,\cdots ,N \end{array}$$ (5)

The age of all particles is set to zero during the initialization. The carried states of the particles are assigned a small variance that represents the uncertainty of the wind measurement and temperature:

$$\begin{array}{c} (\begin{array}{c} u_{p,i}\\ v_{p,i}\\ \tau_{p,i} \end{array})∼N\;([\begin{array}{c} u\\ v\\ \tau \end{array}],[\begin{array}{ccc} \sigma_{u0}^{2} & 0 & 0\\ 0 & \sigma_{v0}^{2} & 0\\ 0 & 0 & \sigma_{\tau 0}^{2} \end{array}])\;i=1,2,\cdots ,N \end{array}$$ (6)

**Fig 4:**
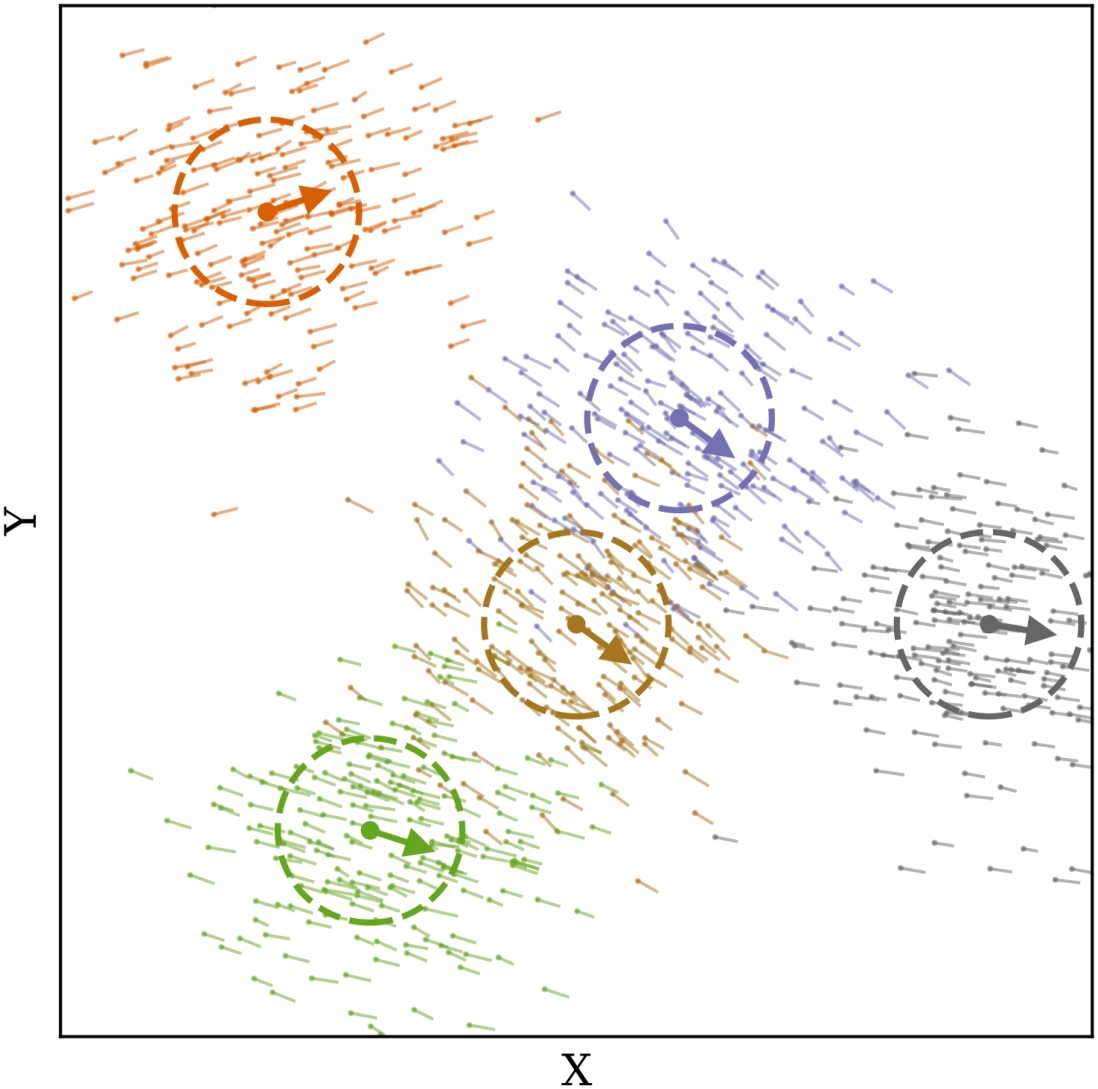
Wind measurements and particle initialization.

As an example, Fig 4 displays the wind vectors in solid arrows, as well as the generated particles in thin vectors (after the first propagation step). The plot shows the 2D projection of the X-Y plane, and only a small percentage of all particles are illustrated. The dashed circles indicate the variance of particle positions in relation to the measurement location.

### 3.4 Particle propagation

Particle motion follows a Gaussian random walk model. At each update step, the particle age (*α*) increases. The following equation describes the motion model of a particle.

$$\begin{array}{c} \begin{array}{cc} \left(\begin{array}{c} x_{p,i,t+1}\\ y_{p,i,t+1}\\ z_{p,i,t+1} \end{array}\right) & =(\begin{array}{c} x_{p,i,t}\\ y_{p,i,t}\\ z_{p,i,t} \end{array})+\Delta P_{t}\;i=1,2,\cdots ,N\\ \Delta P_{t} & ∼N([\begin{array}{c} k_{2}u_{p}\\ k_{2}v_{p}\\ 0 \end{array}],[\begin{array}{ccc} \sigma_{px}^{2} & \sigma_{pxy} & 0\\ \sigma_{pxy} & \sigma_{py}^{2} & 0\\ 0 & 0 & \sigma_{pz}^{2} \end{array}]) \end{array} \end{array}$$ (7)

**Fig 5:**
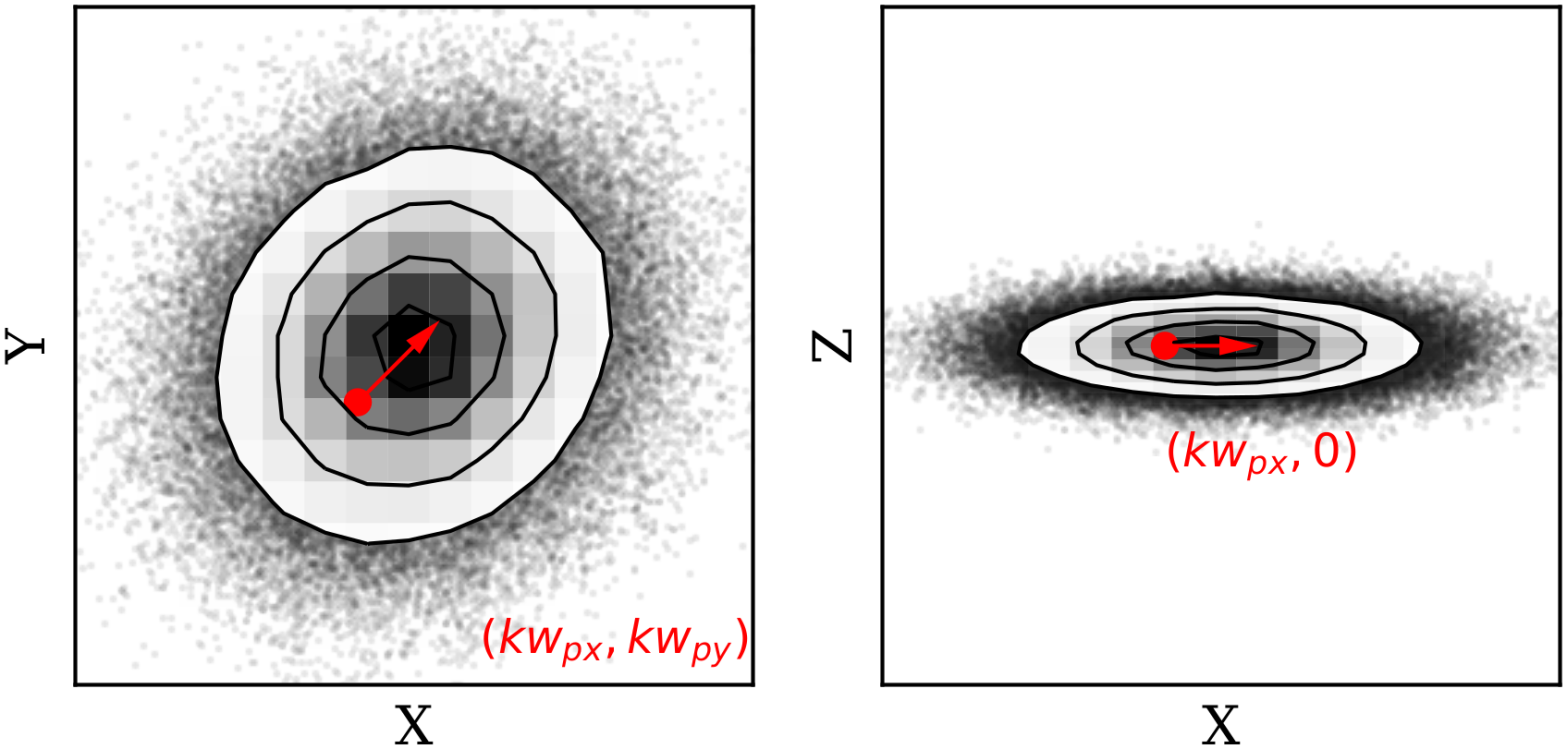
Random update of a particle position.

The step factor Δ*P* is different in horizontal and vertical direction. Horizontally, the terms *k*_2_*u*_*p*_ and *k*_2_*v*_*p*_ allow the random walk to be executed with a small bias along the direction of the wind, with a scaling factor *k*_2_. Choosing a larger *k*_2_ allows the propagation to become more biased toward the wind direction. Vertically, the propagation follows a zero-mean Gaussian walk. The particle motion model is illustrated in Fig 5, where two projections (*X*-*Y* and *X*-*Z*) of a possible particle update are shown. The red dot represents the position (*x*_*p*,*t*_, *y*_*p*,*t*_, *y*_*p*,*t*_), while the probability of the next position (*x*_*p*,*t*+1_, *y*_*p*,*t*+1_, *y*_*p*,*t*+1_) is shown by the contour plot. The vector equals to $E[\Delta P_{t}]$.

**Fig 6:**
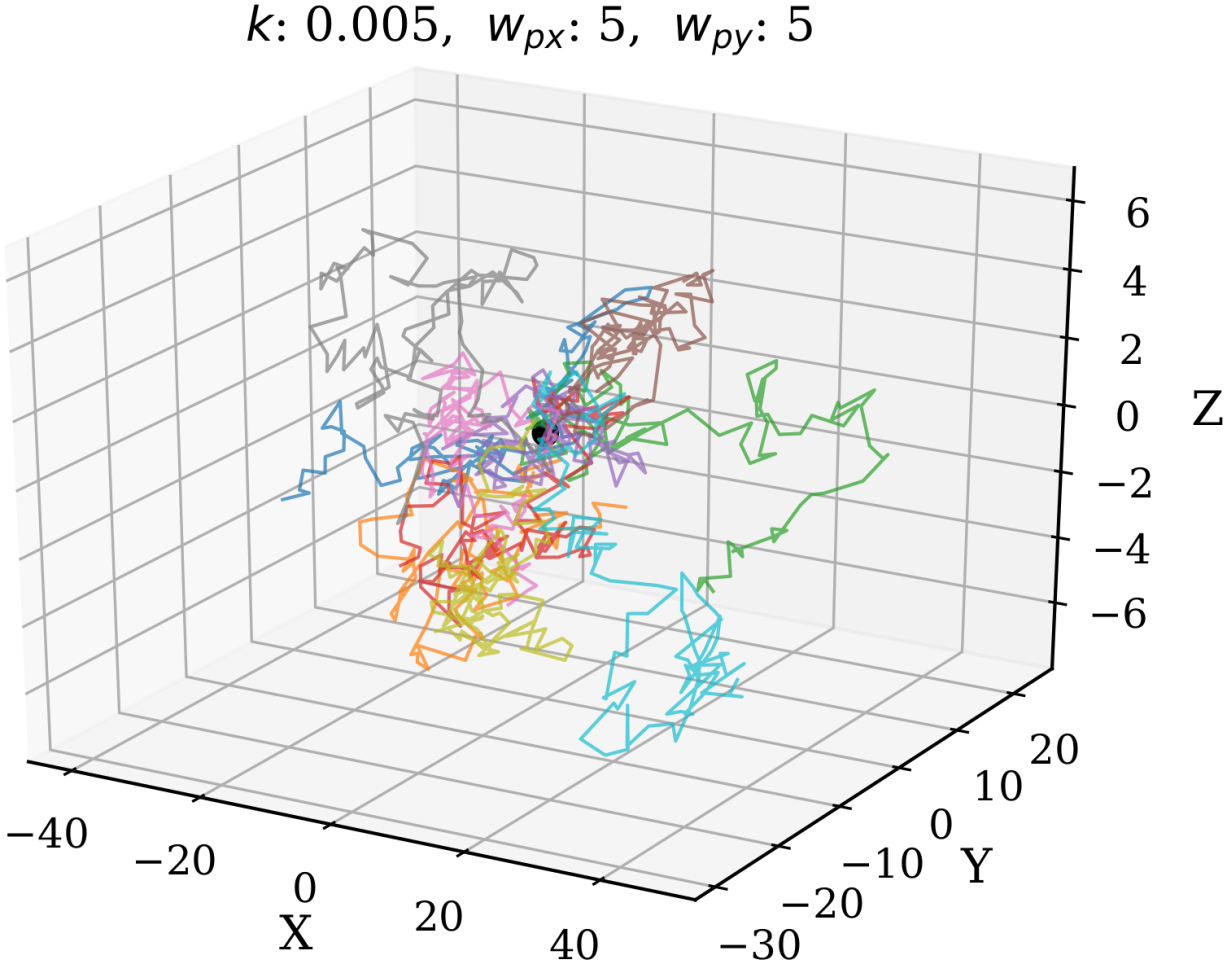
Particle random walks in 3D.

The updates of particles follow the Gaussian random walk as shown in Fig 6, where several possible 100-step random walks of a particle (with origin [0, 0, 0]) are illustrated. Different trajectories are distinguished by different colors.

### 3.5 Probabilistic re-sampling

At the end of each update, the particles are re-sampled. First, all particles that have propagated outside of the horizontal and vertical boundaries are removed. Then all particles are sampled by age according to the probability computed in Eq 8:

$$\begin{array}{c} p\left(\alpha \right)=\text{exp}(-\frac{\alpha^{2}}{2\sigma_{\alpha }^{2}}) \end{array}$$ (8)

where *α* represents the age of the particles and *σ*_*α*_ is the control parameter. This re-sampling maintains the number of particles in the system according to age. There are always more new particles than older particles in the entire system.

### 3.6 Information reconstruction

At any location, the wind and temperature information can be reconstructed by using surrounding particles. Since wind and temperature are distributed differently, different formulas are used to compute their values. Let position (*x*, *y*, *z*) be the location where wind and temperature are to be computed. The wind is constructed using the weighted wind state values from the neighboring particles, denoted as *P*. A particle *p*, with location (*x*_*p*_, *y*_*p*_, *z*_*p*_) is considered in the set *P* if it is within the boundary of

$$\begin{array}{c} \begin{array}{c} x-x_{b}\leq x_{p}\leq x+x_{b}\\ y-y_{b}\leq y_{p}\leq y+y_{b}\\ z-z_{b}\leq z_{p}\leq z+z_{b} \end{array} \end{array}$$ (9)

Subsequently, the wind at location (*x*, *y*, *z*) is computed as the weighted sum of the wind state information carried by the considered particles:

$$\begin{array}{c} \left[\begin{array}{c} u\\ v \end{array}]=\frac{1}{\sum_{p\in P}W_{p}}\cdot \sum_{p\in P}(W_{p}\cdot [\begin{array}{c} u_{p}\\ v_{p} \end{array}]\right) \end{array}$$ (10)

For temperature, we introduce an additional condition to the particle selection in addition to Eq 9 to ensure that the origin of the particles is also at a similar altitude:

$$\begin{array}{c} z-z_{b}\leq z_{p0}\leq z+z_{b} \end{array}$$ (11)

After obtaining the reduced set of particles P*, the computation of the temperature at location (*x*, *y*, *z*) also becomes a weighted sum of the temperatures from the considered particles:

$$\begin{array}{c} \tau =\frac{1}{\sum_{p\in P^{*}}W_{p}}\cdot \sum_{p\in P^{*}}\left(W_{p}\cdot \tau_{p}\right) \end{array}$$ (12)

In Eqs 10 and 12, *W*_*p*_ is the weight of each particle that is computed based on the product of two exponential functions. Function *f*_*d*_(⋅) draws an exponential relationship between weight and distance between the particle and the coordinate where wind and temperature need to be calculated. Function *f*_0_(⋅) defines the weight of the particles and depends on the distance of the particles from their origins.

$$W_{p}=f_{d}\left(d\right)\cdot f_{0}\left(d_{0}\right)$$ (13)

$$f_{d}\left(d\right)=\text{exp}(-\frac{d^{2}}{2C_{d}^{2}})$$ (14)

$$f_{0}\left(d_{0}\right)=\text{exp}(-\frac{d_{0}^{2}}{2C_{0}^{2}})$$ (15)

Here, *d* represents the spatial distance between a particle and the location of interest. *C*_*d*_ and *C*_0_ are control parameters for the functions *f*_*d*_(⋅) and *f*_0_(⋅).

**Fig 7:**
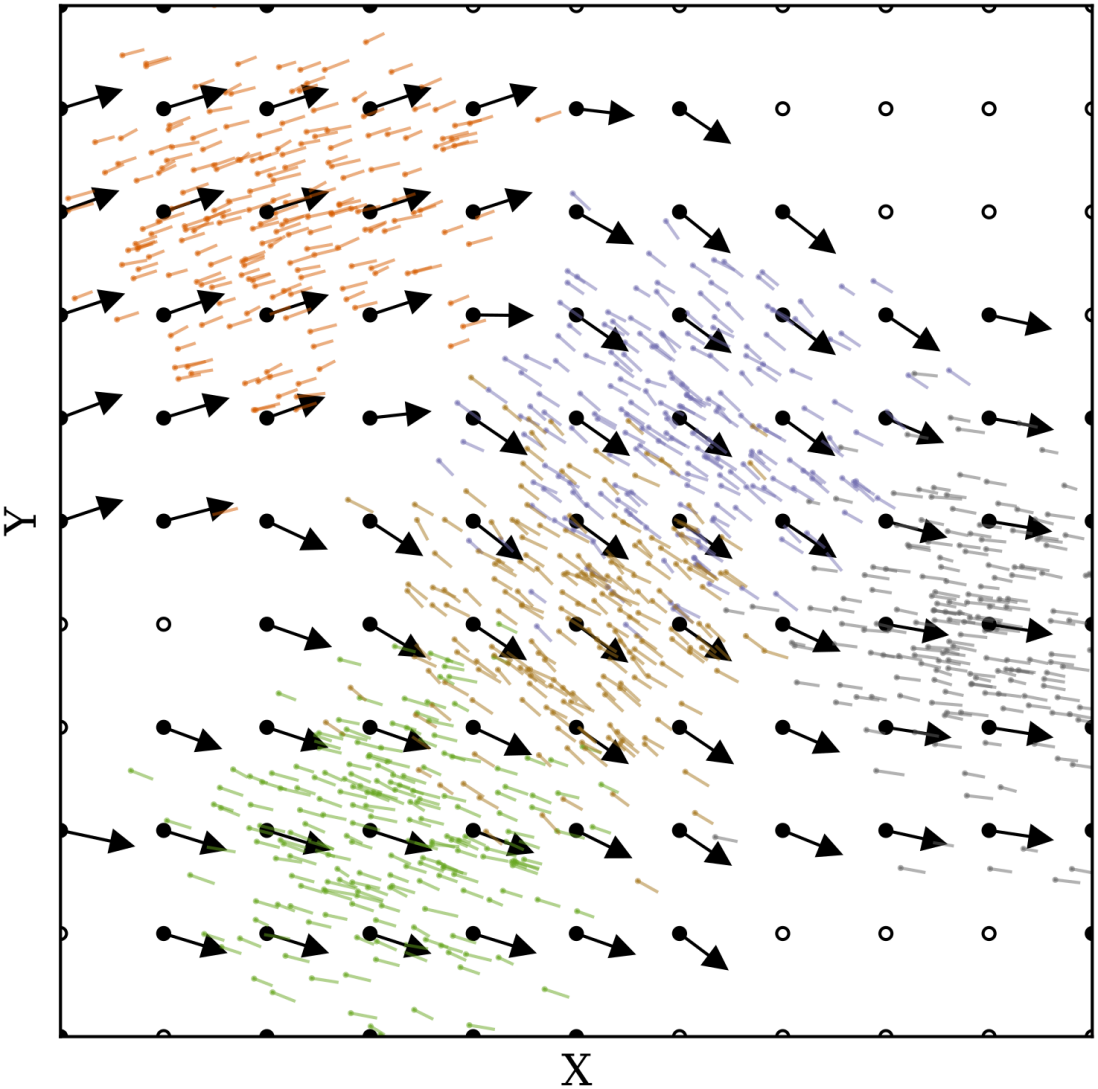
Wind field constructed from particles.

Fig 7 displays a re-constructed wind field from previously generated particles, at time-step zero. At each grid point, the wind vector is shown in solid arrows. Grid points with no information yet are presented in scattered circles.

It is worth pointing out that the MP model does not use a pre-defined grid. Particles are generated as weather measurements and are henceforth propagated independently. Values can be computed at any point or any set of points at the current time using Eq 10, as long as a sufficient number of particles exist in the neighborhood of these locations. In later experiments of this paper, we chose a minimum of 10 particles.

### 3.7 Representation of confidence level

Once wind and temperature are reconstructed, it is also important to evaluate the confidence level of the estimates. The confidence level is computed as the combination of confidence functions that are based on several independent factors. These factors are:

1. the number of particles in the vicinity of the location of interest (*N*)
2. the mean distances between particles and the location of interest (*D*)
3. the homogeneity of states carried by particles (*H*)
4. the strength of the particles due to aging function (*S*)

#### 3.7.1 Particle numbers and distances

The number of particles *N* represents the number of particles that are present within the calculation area. The mean distance to the point of interest is denoted as *D*. Higher confidence values are assigned to locations where more particles are present nearby. Areas that are far from flight trajectories tend to have fewer reachable particles and should yield lower confidence values.

#### 3.7.2 Homogeneity of carried states

The level of homogeneity refers to the similarity of particle states. It essentially indicates whether different measurements propagated from a nearby area indicate similar evidence of wind vectors. The homogeneity of wind (*H*_*w*_) is computed as the spectral norm of the covariance matrix of the wind vector components of the particles. For the temperature state, the homogeneity *H*_*τ*_ is simply represented by the variance of the temperature of the particles:

$$\begin{array}{c} \begin{array}{cc} \Sigma & =Cov(u_{p},v_{p})\\ H_{w} & ={∥\Sigma ∥}_{2}=\sqrt{\lambda_{max}\left(\Sigma^{T}\Sigma \right)}\\ H_{\tau } & =\text{Var}(\tau_{p}) \end{array} \end{array}$$ (16)

Here, λ_*max*_ represents the largest eigenvalue of a matrix, and ***u***_*p*_, ***v***_*p*_, and ***τ***_*p*_ are wind and temperature states of the particles within the computing bound.

#### 3.7.3 Particle strength

From the creation of a particle, its age (*α*) increases at each step of propagation. Since the particles are sampled at each update according to age, the strength parameter can simply be calculated as the fraction of the mean particle ages.

$$\begin{array}{c} S=\frac{1}{{\bar{\alpha }}_{p}} \end{array}$$ (17)

#### 3.7.4 Normalized and combined confidence

Values from all four confidence factors have a distinct range. It is important to normalize these factors into the same range. Linear scaling is applied to convert all values of each factor into the (0, 1) range.

$$\begin{array}{c} s\left(x\right)=\frac{x-\text{min}(X)}{\text{max}(X)-\text{min}(X)} \end{array}$$ (18)

At any given time, the confidence vectors that represent all wind grid points are *N*, *D*, *H*, and *S*. Then, the combined confidence is expressed as:

$$\begin{array}{c} \begin{array}{cc} C_{w} & =\text{mean}\{s\left(N\right),s\left(D\right),s\left(H_{w}\right),s\left(S\right)\}\\ C_{\tau } & =\text{mean}\{s\left(N\right),s\left(D\right),s\left(H_{\tau }\right),s\left(S\right)\} \end{array} \end{array}$$ (19)

**Fig 8:**
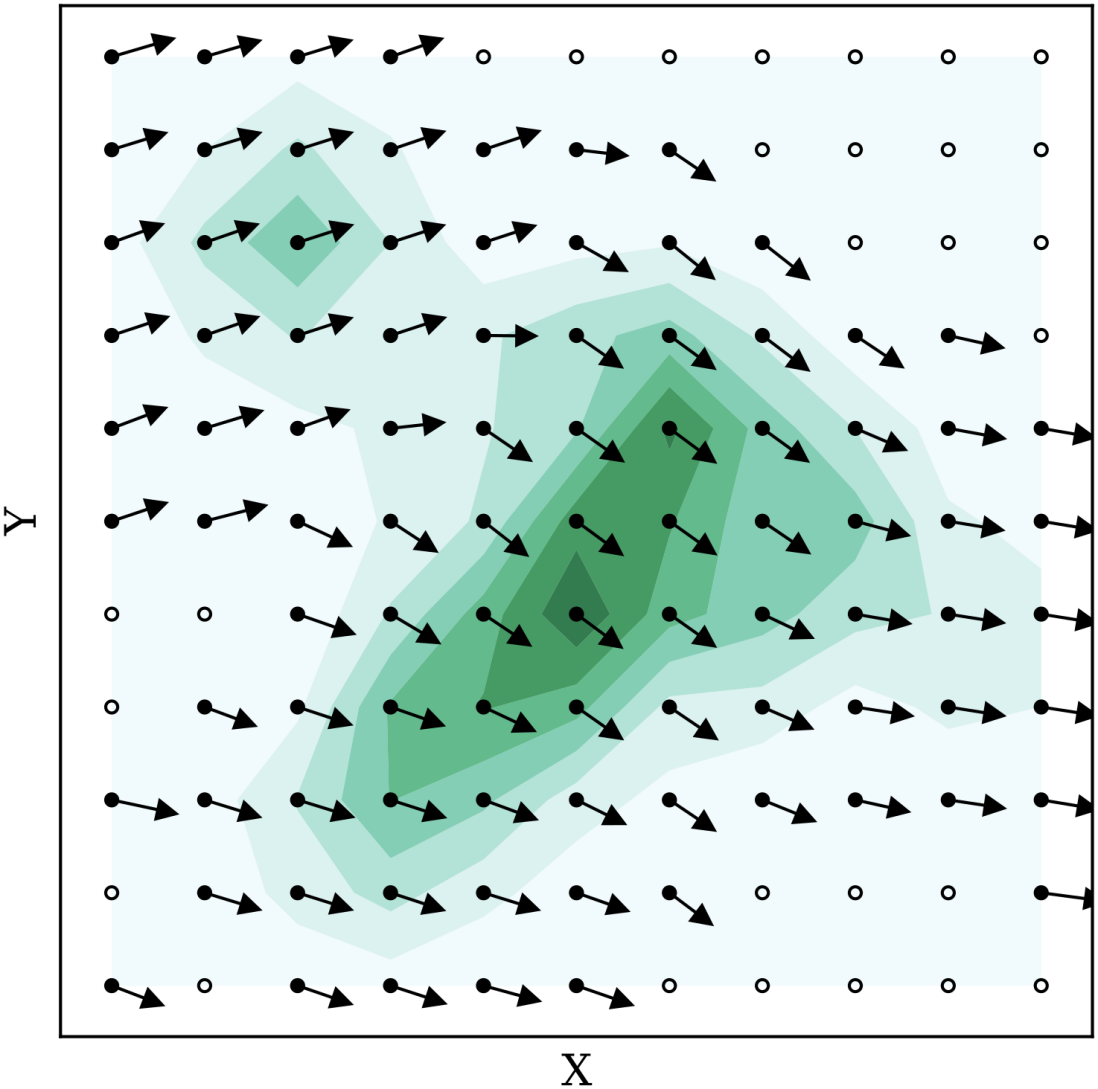
Wind field construction with confidence plot.

Fig 8 illustrates the confidence contour plot based on previously defined calculations. Areas in the plot in darker colors represent higher levels of confidence.

The confidence indicator is a relative value that can be compared within the field at any time instance, but it is not comparable between different time steps due to the normalization undertook during its calculation.

## 4 Short-term prediction with the Meteo-Particle model

We can construct a predictor of wind (u, v component) or temperature as a univariate regression function that is based on particles in the MP model. The regression predictor requires the construction of a statistical model that is a function of time. For each location, surrounding particles are grouped by age, and the means are computed for each existing time step. Thus, we have the input data ***t*** and ***y***_*t*_.

In previous research, Gaussian process regression (GPR) has been used in a similar fashion for the interpolation of wind condition [16, 24]. A similar approach can be applied in the proposed MP model for short-term predictions. From a Bayesian perspective, Gaussian process regression (also know as kriging in geostatistics) is an interpolation method. It can be considered as a form of Gaussian process prediction, which is based on a prior over functions and fitted over the observed data [25].

One way to understand the Gaussian process regression is to view functions as infinite-length vectors. The Gaussian process describes joint distributions over these infinite dimension vectors. The Gaussian process regression considers functions to be drawn from a prior that is defined by a mean function and a kernel (covariance) function. The prior can be formulated as follows:

$$\begin{array}{c} f\left(t\right)=N\{\mu \left(t\right),K\left(t,t^{\prime }\right)\} \end{array}$$ (20)

where *t* and *t*′ are two time instances. *μ*(*t*) and *K*(*t*, *t*′) are the mean and kernel functions respectively. Commonly, the mean function is assumed to be zero (*μ*(***t***) = 0). The kernel function can be chosen from a wide range of options (see Chapter 4 in [26]. The kernel function defines the underlying property of the function *f*(***t***). For example, it can act as a constant component or represent the smoothness and periodic properties. Different types of kernels can be combined as summation or multiplication. In this paper, we use a summation of three kernels to describe the covariance function. Denoting *K*_*C*_, *K*_SE_, and *K*_WN_ as constant kernel, squared exponential kernel, and white noise kernel, the combined kernel is defined as:

$$\begin{array}{c} \begin{array}{cc} K(t,t^{\prime }) & =K_{\text{SE}}\left(t,t^{\prime }\right)+K_{C}\left(t,t^{\prime }\right)+K_{\text{GN}}\left(t,t^{\prime }\right)\\ K_{\text{SE}}\left(t,t^{\prime }\right) & =\sigma_{\text{se}}^{2}\;\text{exp}\{-\frac{1}{2\ell^{2}}{\left(t-t^{\prime }\right)}^{2}\}\\ K_{C}\left(t,t^{\prime }\right) & =C\\ K_{\text{WN}}\left(t,t^{\prime }\right) & =\sigma_{\text{wn}}^{2}\delta \left(t-t^{\prime }\right) \end{array} \end{array}$$ (21)

where *σ*_se_, *ℓ*, *C*, and *σ*_se_ are hyper-parameters for the kernel function. Denoting Θ = {*σ*_se_, *ℓ*, *C*, *σ*_se_} as the vector of hyper-parameters, we can compute the optimal Θ by maximizing the log marginal likelihood function:

$$\begin{array}{c} \begin{array}{cc} \text{log}\;p(y_{t}|t,\Theta ) & =\frac{1}{2}y_{t}^{T}K{\left(t,t\right)}^{-1}y_{t}-\frac{1}{2}\text{log}\;\left|K\left(t,t\right)\right|-\frac{n}{2}\text{log}\;2\pi \\ \hat{\Theta } & =\underset{\Theta }{\text{argmin}}\{\text{log}\;(y_{t}|t,\Theta )\} \end{array} \end{array}$$ (22)

**Fig 9:**
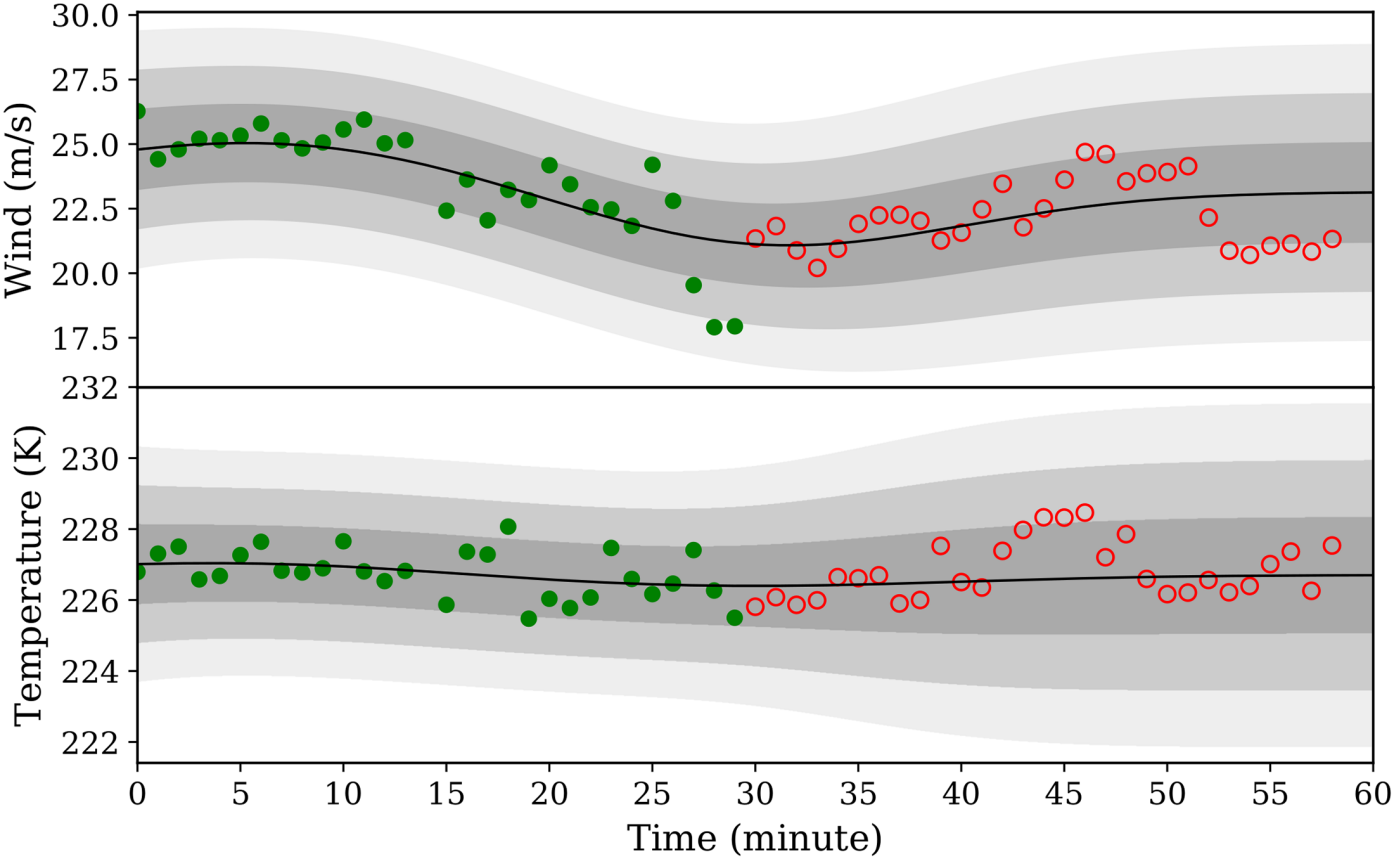
Gaussian process regression predictor example.

Once an optimal set of hyper-parameters is obtained, the probabilistic prediction of future states can be computed as follows:

$$\begin{array}{c} \begin{array}{cc} p(f\left(t^{*}\right)|t^{*},y_{t},t) & =N\{A,B\}\\ A & =K\left(t^{*},t\right)K{\left(t,t^{\prime }\right)}^{-1}y_{t}\\ B & =K\left(t^{*},t\right)-K\left(t^{*},t\right)K{\left(t,t^{\prime }\right)}^{-1}K{\left(t^{*},t\right)}^{T} \end{array} \end{array}$$ (23)

where t* is an unseen or future time instance. The training and prediction require computational expensive inversion of *K*(*t*, *t*′), which can be calculated using Cholesky factorization (see Chapter 2.3 in [26]). The computational complexity of Gaussian regression is *O*(*n*^3^). In Fig 9, using the GPR predictor, a 30-minute prediction based on a 30-minute observation of one location is illustrated.

In this figure, green dots are observations computed using the MP model for the first 30 minutes. The black line is the mean prediction of the GPR model for the entire hour. From darker to lighter color gray areas are *σ*, 2*σ*, and 3*σ* of prediction coverage respectively. The red dots are test observations computed for the second 30 minutes, which are not used for constructing the GPR predictor. In this specific example, we can see that the majority of the estimates in the second 30 minutes are within one *σ* of the prediction interval.

The GPR predictor can be constructed at any position of interest to provide short-term predictions. However, a short history of estimated states from the MP model needs to be recorded for the predictions.

## 5 Experiments and validations

**Fig 10:**
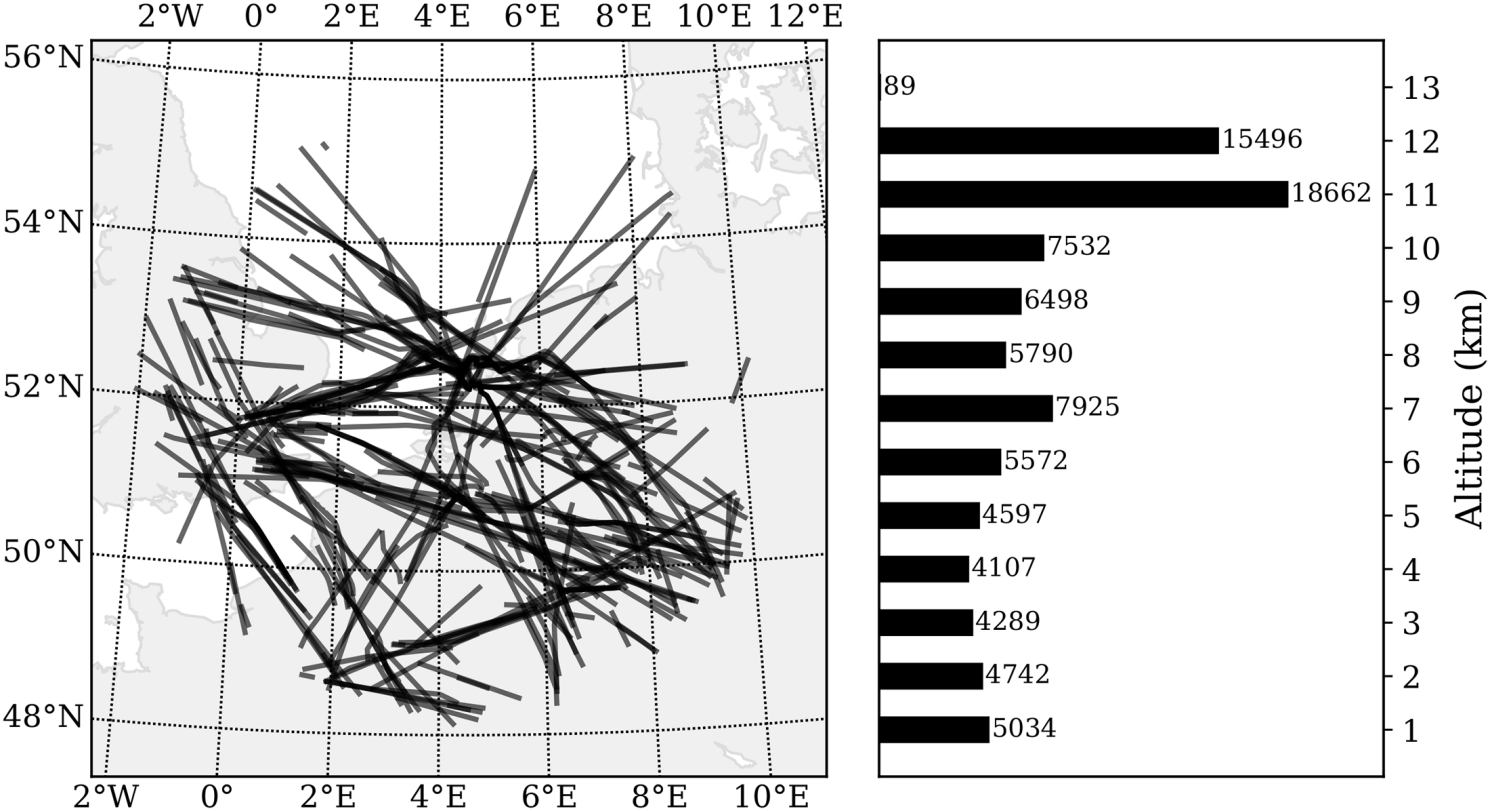
Wind data ground projections and vertical distributions.

An ADS-B/Mode-S receiver is installed at the faculty of Aerospace Engineering at the Delft University of Technology. This device provides a constant stream of signals obtained from aircraft that are within line of sight of this receiver. Using the open-source decoding library pyModeS, ADS-B and Mode-S Comm-B replies can be used to derive wind observations for the MP model. The area of the experiment is between 300 to 400 kilometers in radius, centered around Delft, the Netherlands, as shown in Fig 10.

At first, to demonstrate the Meteo-Particle model, a small dataset (with 30 minutes data) from ADS-B and Mode-S is used to compute the wind observations. The observations are used to construct the wind and temperature fields. The results of the wind and temperature fields are illustrated. Later on, to validate the MP model, we compare the results with two public numerical weather prediction datasets, which are the Global Forecast System (GFS) reanalysis data and the European Centre for Medium-Range Weather Forecast (ECMWF) ERA5 reanalysis data.

### 5.1 Constructing sample wind and temperature fields

**Fig 11:**
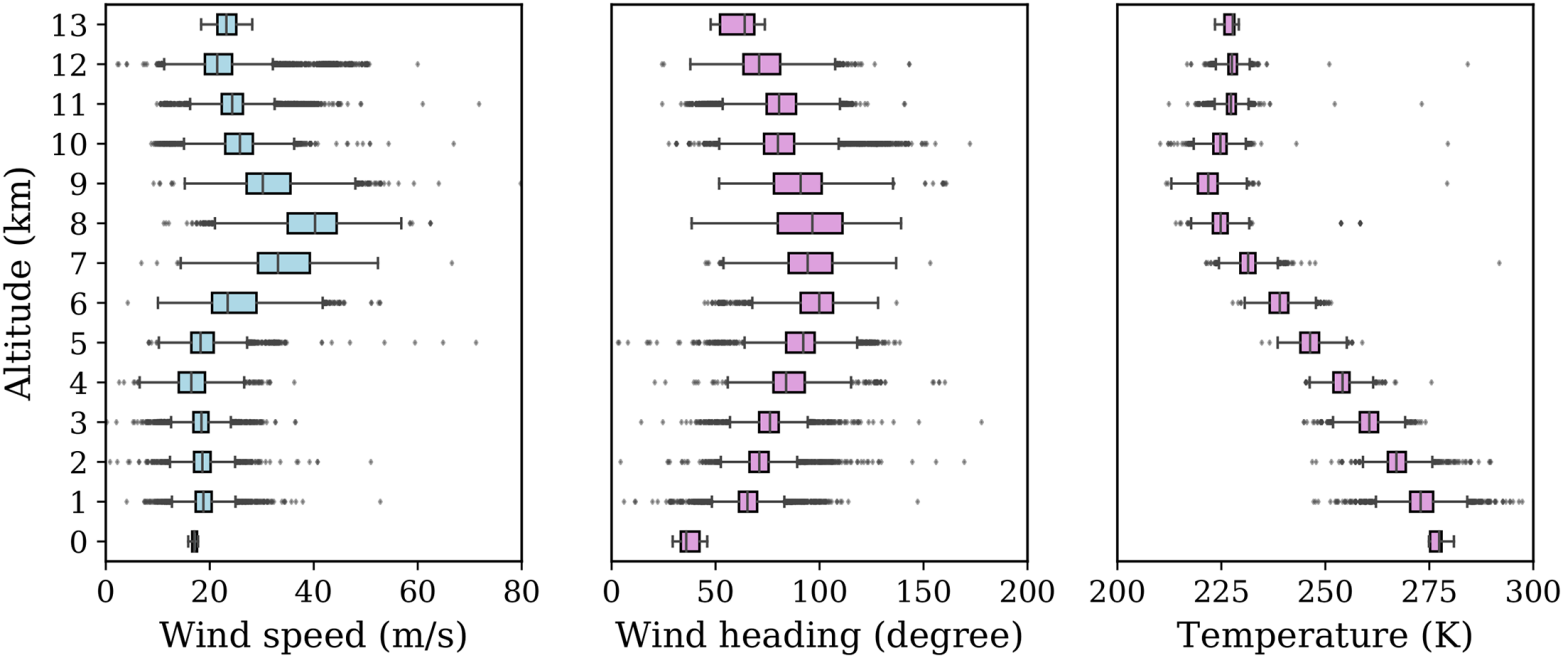
Wind speed and direction distributions grouped by altitude.

As a first experiment, we want to reconstruct the real wind and temperature grid, based on a small set of aircraft surveillance data. The dataset consists of 30 minutes of obtained wind data, from 9:00 to 9:30 UTC on January 01, 2018. In total, around 90,000 wind measurements were generated during this time period. In Fig 10, the distributions of wind observations are displayed both horizontally and vertically. The plot on the left-hand side illustrates the ground projection of all observations. On the right-hand side, the plot shows a histogram with the number of observations per 1 km altitude. It is apparent that, horizontally, the measurements are highly concentrated along flight routes. Vertically, the majority of the observations are at cruise altitudes and lower approaching altitudes. The statistic of wind at different altitudes in this dataset is computed. The distributions grouped by altitude are shown in Fig 11.

During this time period, it can furthermore be observed that wind generally comes from a westerly or south-westerly direction. The spatiotemporally varying wind also leads to variability in both wind velocity and direction even within this short period of time. Figs 10 and 11 reflect the two challenges proposed earlier in this paper. They are 1) highly non-uniformly distributed and varying observation data, and 2) the observation errors (shown as outliers).

**Fig 12:**
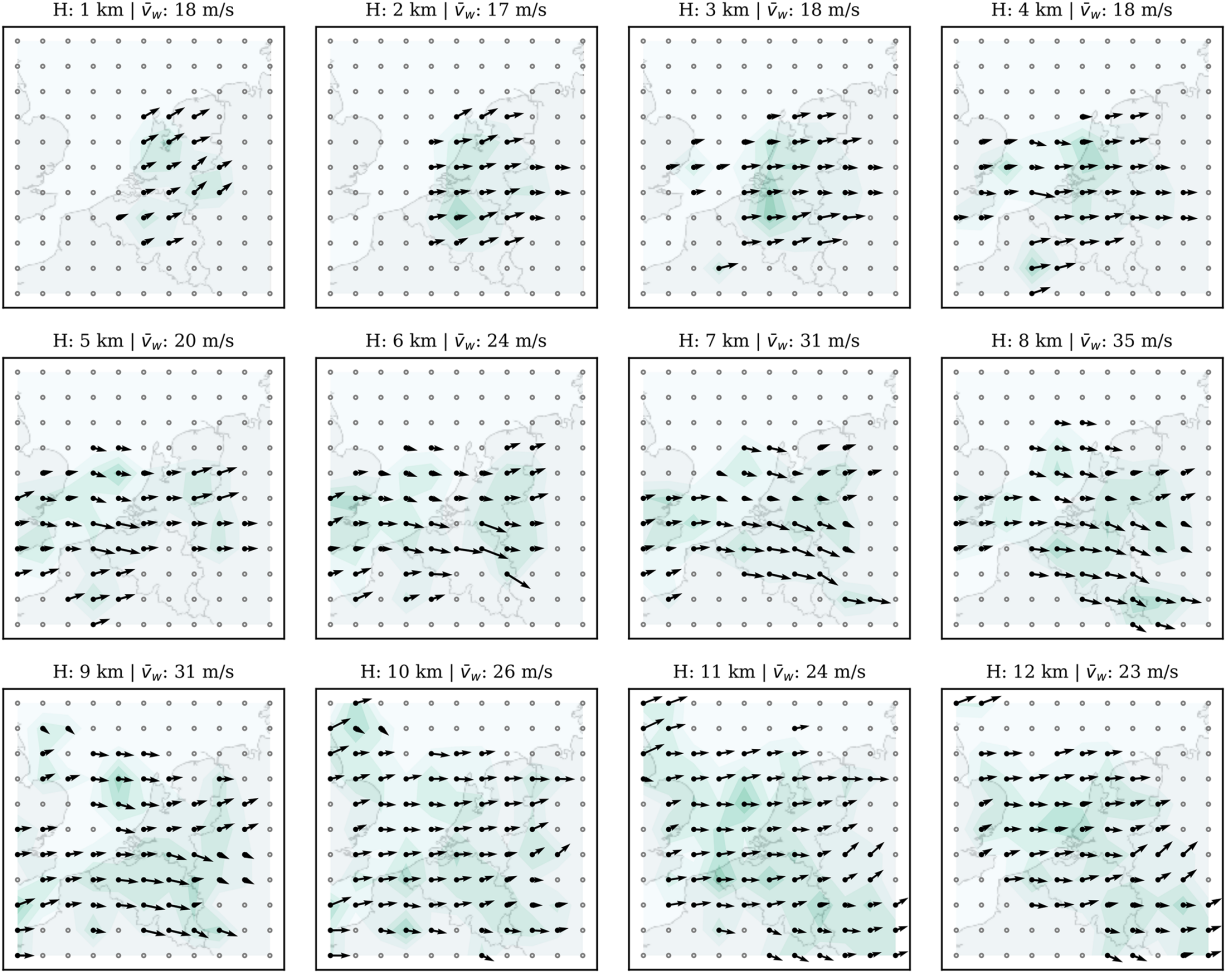
Wind grid at 12 different altitude levels.

**Fig 13:**
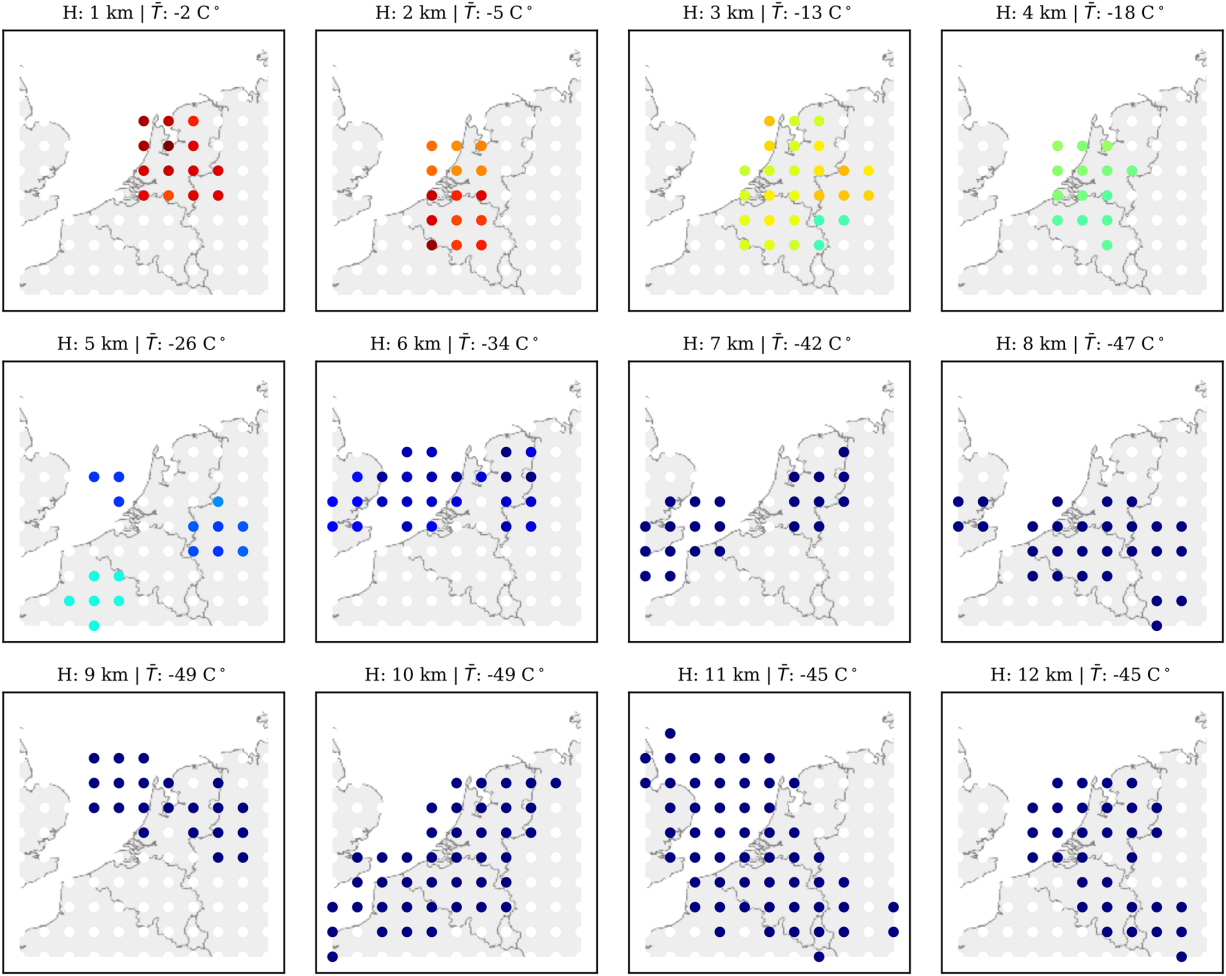
Temperature grid at 12 different altitude levels.

To simulate a real-time run of the model, these recorded wind data are streamed to the MP model using the original sequence based on the data time-stamp. A snapshot of the wind grid at 09:00 UTC is shown in Fig 12, while the temperature grid is shown in Fig 13. In these two figures, the entire airspace is represented by a field consisting of 10 x 10 x 12 grid-points. It is centered around the location of the receiver (latitude: 51.99°N, longitude: 4.37°E). The horizontal grid spacing is approximately 60 km. Vertical grid spacing is 1 km. We can observe visually that both wind and temperature are consistent with the observation distributions from Fig 11.

### 5.2 Validation of MP model with NWP data

In this section, we focus on validating the correctness of the MP model output. The level of correctness can be examined against data from existing meteorological models. GFS reanalysis data and ECMWF ERA5 reanalysis data are used to examine the MP model result. A period of 10 days (from the 1st to the 10th of January 2018) is used in this experiment.

GFS reanalysis data provide global atmospheric reanalysis of all altitudes at the highest available resolution of 0.5 degree in latitude and longitude. Meteorological conditions (including wind and temperature) are computed at 00:00, 06:00, 12:00, and 18:00 hour each day. The ECMWF ERA5 can provide higher resolution reanalysis data. We use the 0.25 degree resolution data as the point of comparison.

The wind and temperature observations are computed based on aircraft surveillance data, which contains 10 days of one-hour data around the four hours indicated in GFS reanalysis grid data.

At each GFS hour (00:00, 06:00, 12:00, or 18:00), spot values and average values are computed. The spot value is the wind grid computed from the MP model at the exact GFS hour. The average values are computed as the mean of the hour around GFS hour (per minute, +/- 30 minute of wind grids). Spatially, we extract wind/temperature fields at the grid indicated in the GFS and ECMWF ERA5 data, with the resolution of 0.5 and 0.25 degrees respectively.

In order to compare the difference in wind vectors between MP model and NWP data, two distance matrices are used, which are *angular difference* and *magnitude difference*. The angular difference is computed as follows:

$$\begin{array}{c} \Delta \theta =\text{arccos}(\frac{V_{\text{pm}}\cdot V_{\text{gfs}}}{∥V_{\text{pm}}∥\cdot ∥V_{\text{gfs}}∥}) \end{array}$$ (24)

where *V*_pm_ and *V*_gfs_ are the two wind vectors computed by the MP model and extracted from GFS respectively. Δ*θ* is is the angle in degrees between two wind vectors with a range of [0, 180]. The smaller the Δ*θ*, the smaller the angular difference between the two wind vectors. The magnitude difference is computed as the absolute difference of wind vectors:

$$\begin{array}{c} \Delta V=\text{abs}(∥V_{\text{pm}}∥-∥V_{\text{gfs}}∥) \end{array}$$ (25)

Similarly, the temperature difference is computed as:

$$\begin{array}{c} \Delta T=\text{abs}(T_{\text{pm}}-T_{\text{gfs}}) \end{array}$$ (26)

**Fig 14:**
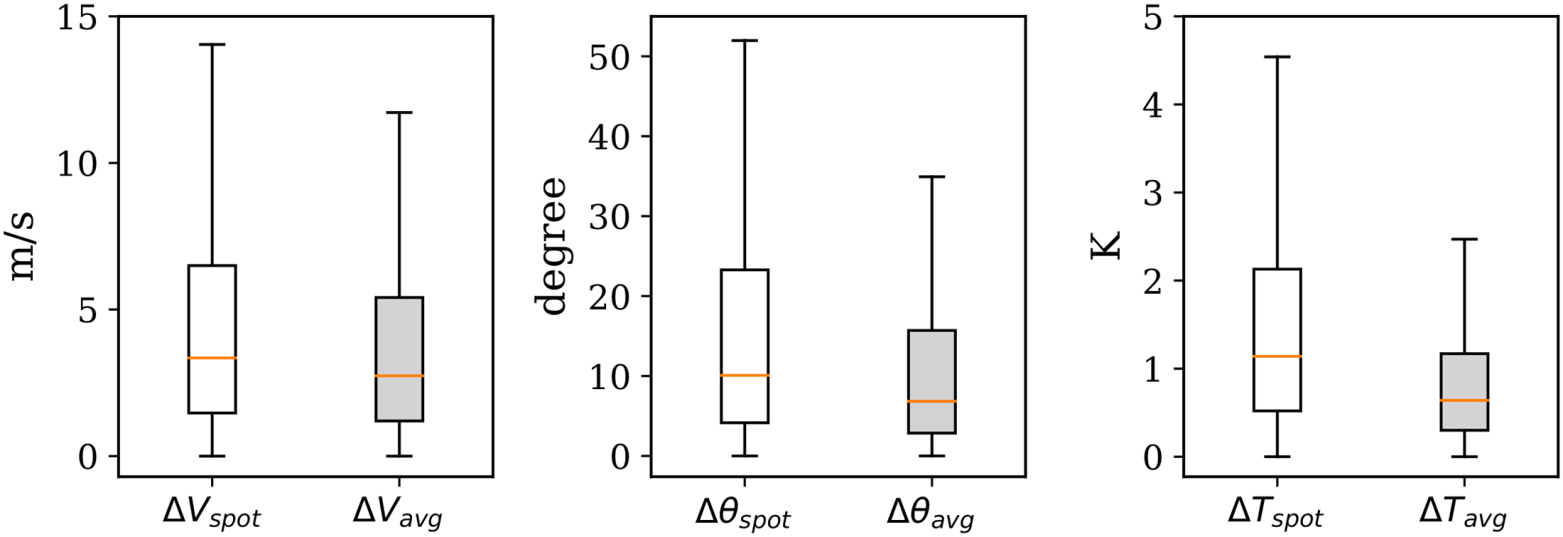
Mean wind and temperature difference with GFS reanalysis data, with 0.5 degree resolution (outliers of boxplots not shown).

**Fig 15:**
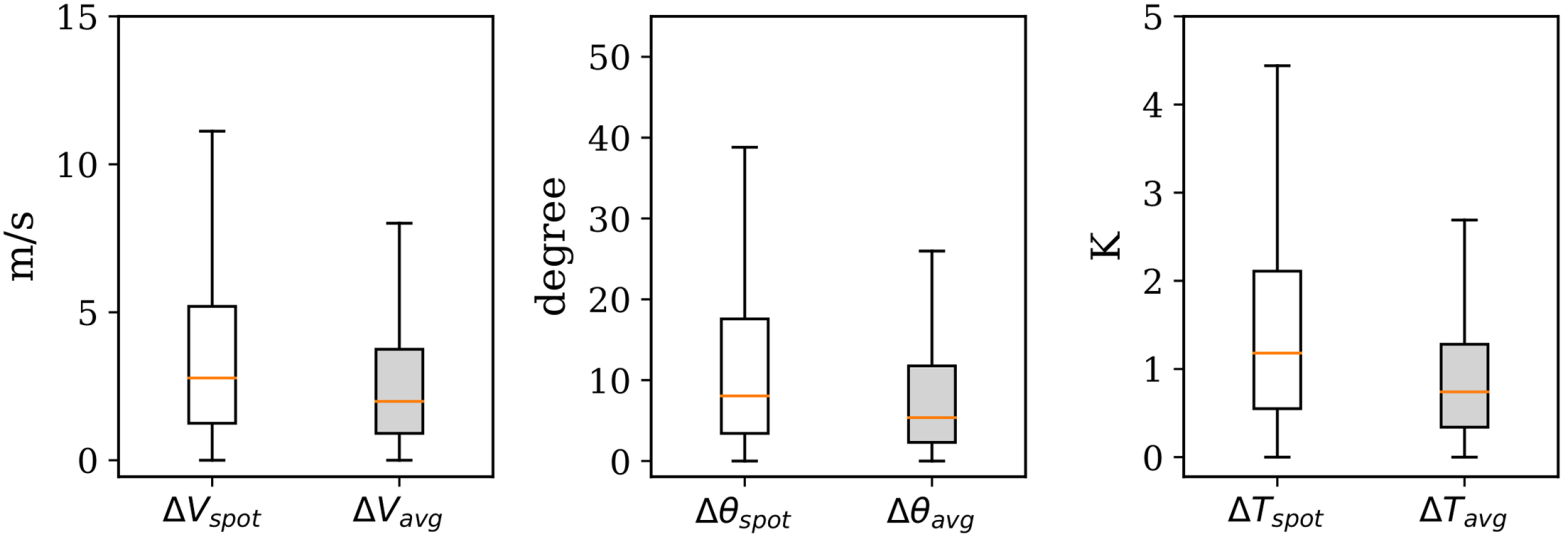
Mean wind and temperature difference with ECMWF ERA5 reanalysis data, with 0.5 degree resolution (outliers of boxplots not shown).

The spot value and average value are first computed using the MP model. The difference with the GFS data is illustrated in Fig 14, showing the result of a total of around 62,000 point comparisons during the 10-day period. The difference between the MP result and the ECMWF data is illustrated in Fig 15. The statistics are computed based on around 300,000 data points due to the higher grid resolution. In each figure, the three plots represent the differences in wind magnitude, wind direction, and temperature respectively.

We can see that the result of the MP model is closer to the higher resolution ECMWF ERA5 data. The exact differences are summarized in Table 2. It can also be observed that when using a one-hour average (mean of 60 snapshots on each minute), the differences become smaller. This would reflect the averaging effects of NWP models. It would also be caused by the lack of interpolation accuracy due to the gaps in source data of these NWP models.

**Table 2:** Comparing MP with NWP models (mean absolute errors).

	Δ*V*_*spot*_	Δ*V*_*avg*_	Δ*θ*_*spot*_	Δ*θ*_*avg*_	Δ*T*_*spot*_	Δ*T*_*avg*_
GFS (0.5°)	3.35	2.74	10.09	6.84	1.14	0.64
ECWMF (0.25°)	2.78	1.99	8.05	5.37	1.18	0.74

### 5.3 MP model estimation accuracy

Accuracy poses one of the common drawbacks of using an NWP model (such as the previously used GFS) for aircraft performance studies. Due to the fixed grid and the large update time interval, interpolation models generated based on an NWP analysis dataset are often over-smoothed. In the low-resolution meteorological dataset, local variations are often absent. When studying aircraft performance, accurate information on the local wind condition is important and sometimes critical. In this experiment, our goal is to study the accuracy of the proposed MP model compared to the interpolated model from NWP analysis data. The same NWP data source, the Global Forecast System reanalysis data (0.5° resolution), is used for this purpose.

To compare the accuracy of the two models, we use the same set of the 30-minute wind data as shown in the earlier section. The approximately 90,000 data points are split in training (60%) and testing (40%) datasets randomly.

To examine the accuracy of the MP model estimation, we use the training dataset to construct the wind and temperature fields using the MP model. Then we estimate the wind and temperature for all points (4D) that appear in the test set. The estimations and actual values are compared to calculate the accuracy metrics. To calculate the accuracy metrics based on NWP data, a linear interpolation model is generated using two GFS analysis datasets (06:00 and 12:00 UTC). In this way, we can compute the wind and temperature of each point that appears in the test dataset.

**Fig 16:**
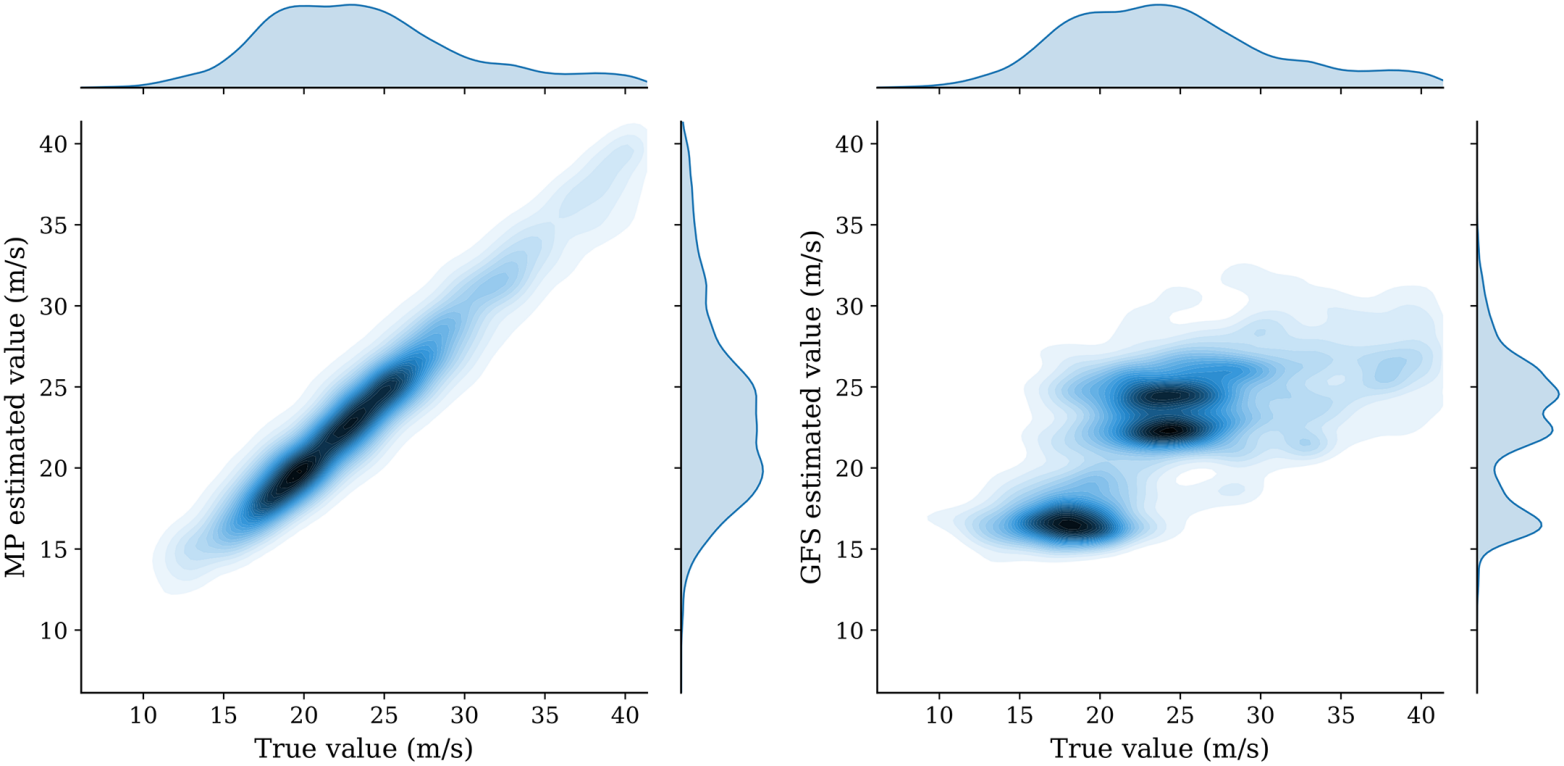
Comparision of MP and GFS model wind prediction.

**Fig 17:**
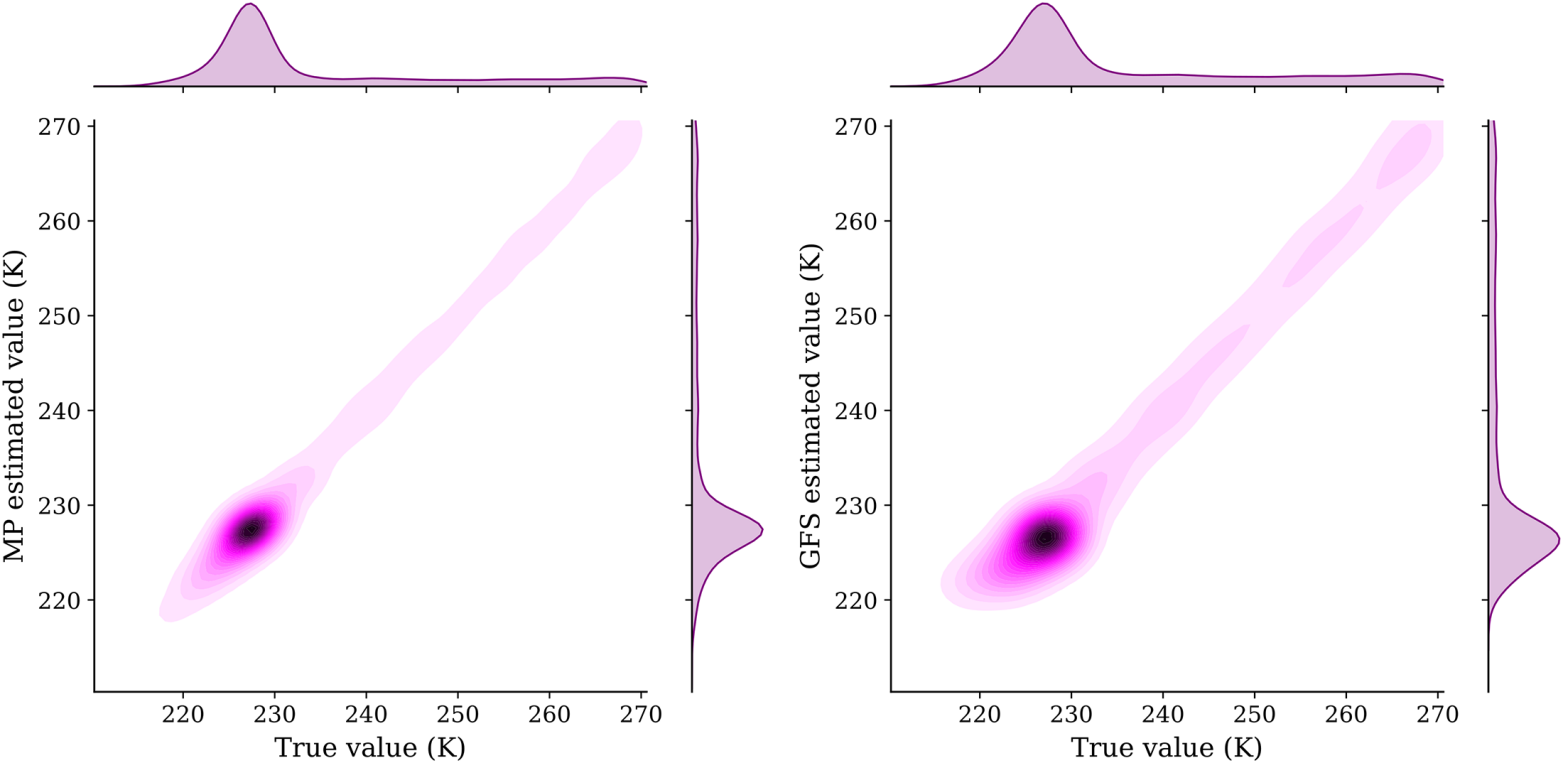
Comparision of MP and GFS model temperature prediction.

Results from two estimation models are compared with the true value in the test dataset. They are illustrated in Fig 16 (wind speed) and Fig 17 (temperature).

We can see that the MP model displays a significantly higher accuracy than the GFS interpolation model when inferring the wind. In terms of temperature, the MP model also shows better accuracy. To quantify the differences, different training/testing splits are chosen for the prediction. Quantitative metrics *Mean Squared Error* (MSE) and *Mean Absolute Error* (MAE) are computed for wind and temperature under each configuration. The results are shown in Table 3 (wind speed) and Table 4 (temperature).

**Table 3:** Estimation errors in wind calculation (m/s).

Train-Test split	MSE(GFS)	MAE(GFS)	MSE(MP)	MAE(MP)
60%-40%	27.64	3.92	3.69	1.30
70%-30%	27.60	3.92	3.71	1.30
80%-20%	28.46	3.98	3.63	1.29
90%-10%	29.05	4.03	3.26	1.26

**Table 4:** Estimation errors in temperature calculation (K).

Train-Test split	MSE(GFS)	MAE(GFS)	MSE(MP)	MAE(MP)
60%-40%	4.81	1.63	3.11	1.20
70%-30%	4.82	1.63	3.00	1.18
80%-20%	4.76	1.62	2.93	1.17
90%-10%	4.75	1.61	2.87	1.14

From the above results, when comparing the absolute error, we can conclude that the wind error decreases around 67%, from around 4 m/s in the GFS model to around 1.3 m/s in the MP model. This represents a threefold increase in wind accuracy. At the same time, the temperature error also decreases around 26%, from 1.6K in the GFS model to around 1.2K in the MP model. This represents a 1.3 fold increase in temperature accuracy.

### 5.4 Short-term prediction accuracy

A short-term prediction (up to 30 minutes) is constructed based on the Gaussian process regression predictor defined in Eq 21. The experiment is carried out using the well-established open-source machine learning library Scikit-Learn [27]. It has the implementation of the proposed Gaussian Process Regression models. The validation experiment is base on the 10x10x12 grid (as show in Figs 12 and 13).

A new dataset consisting of 30 minutes from 9:30 to 10:00 UTC on January 01, 2018, is used for testing predictions made. The true wind and temperature values are computed based on this dataset using the MP model. The prediction of 9:31 to 10:00 is performed at 9:30 based on the wind and temperature fields that have been constructed using the MP model.

**Fig 18:**
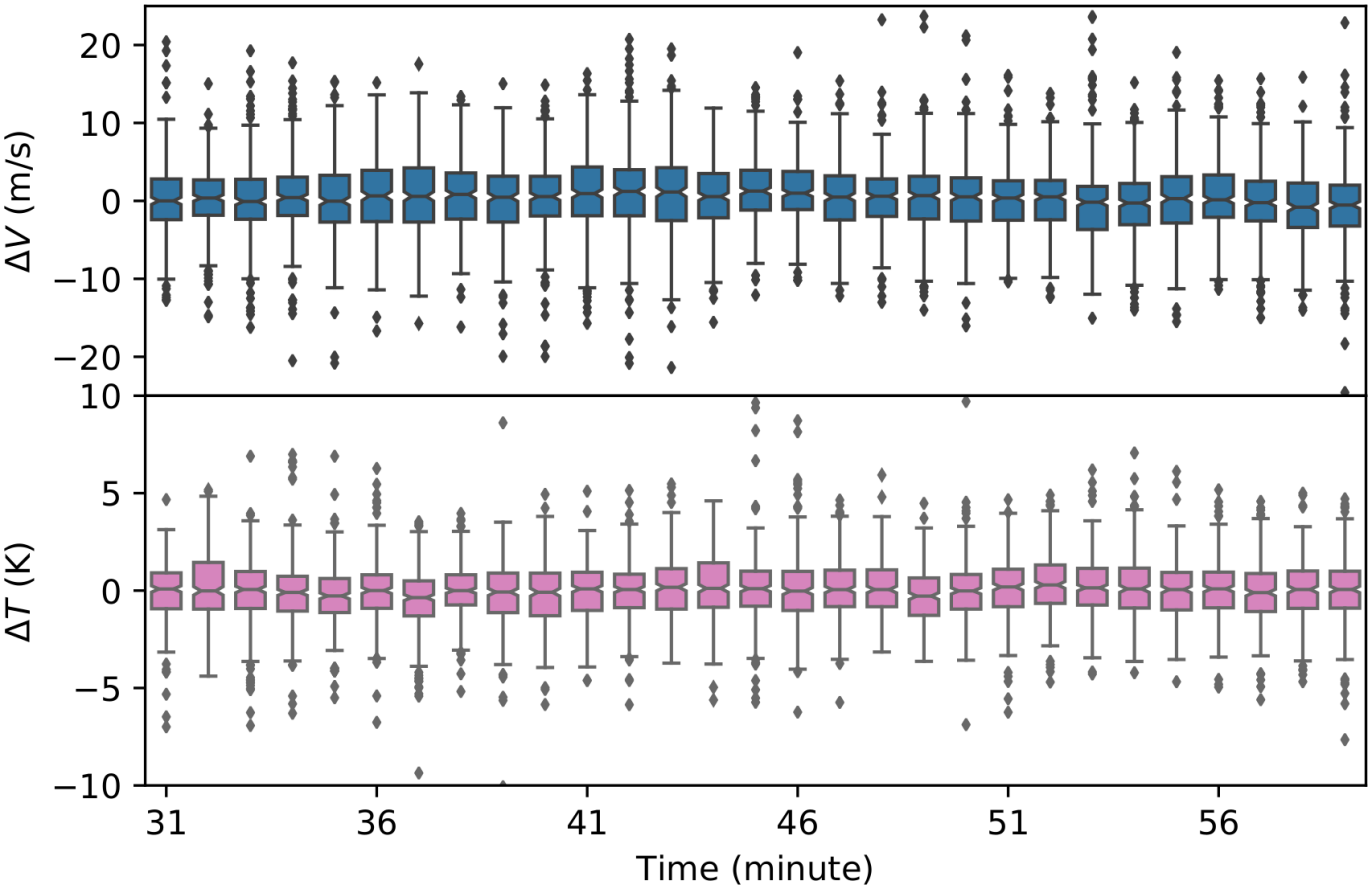
Prediction error of wind and temperature in 30 minutes.

At each minute from 9:31 to 10:00, the difference between the prediction and the true value of all points are plotted in Fig 18.

In this figure, we can see that the differences between true wind and predicted wind are within 10 m/s, whereas the differences of temperatures are within 5 K. The exact error metrics are listed in Table 5.

**Table 5:** Error metrics for the 30 minutes prediction.

	ME	MAE	RMSE
Wind spped (m/s)	0.37	3.71	24.71
Temperature (K)	0.02	1.23	2.80

## 6 Uncertainty analysis

The variability of the system depends on the uncertainty in the MP model and observation data. The uncertainty of the MP model is caused by the stochastic process involved, such as the probabilistic observation rejection and the particle position update. On the other hand, the uncertainties of wind and temperature observations are caused by the inaccurate information downlinked from aircraft, or due to decoding errors.

In this section, we focus on the study of variation caused by the randomness in the stochastic process and data, as well as the errors tolerance of the MP model. All analyses are based on the same dataset used in the earlier experiments, from 9:30 to 10:00 UTC on January 01, 2018, collected by our ADS-B receiver located at Delft, the Netherlands.

### 6.1 Model uncertainty

Randomness exists in the MP model due to the stochastic processes, probabilistic rejection, and sampling. To study whether the combined randomness would affect the wind and temperature field results, as well as the level of the influence, we conduct multiple (100) runs of the model based on the same input data. The wind field at 09:00 (as shown in Fig 12) is measured at the end of each run. Combining all 100 results, we can understand the variation caused by the stochastic elements in the MP model.

**Fig 19:**
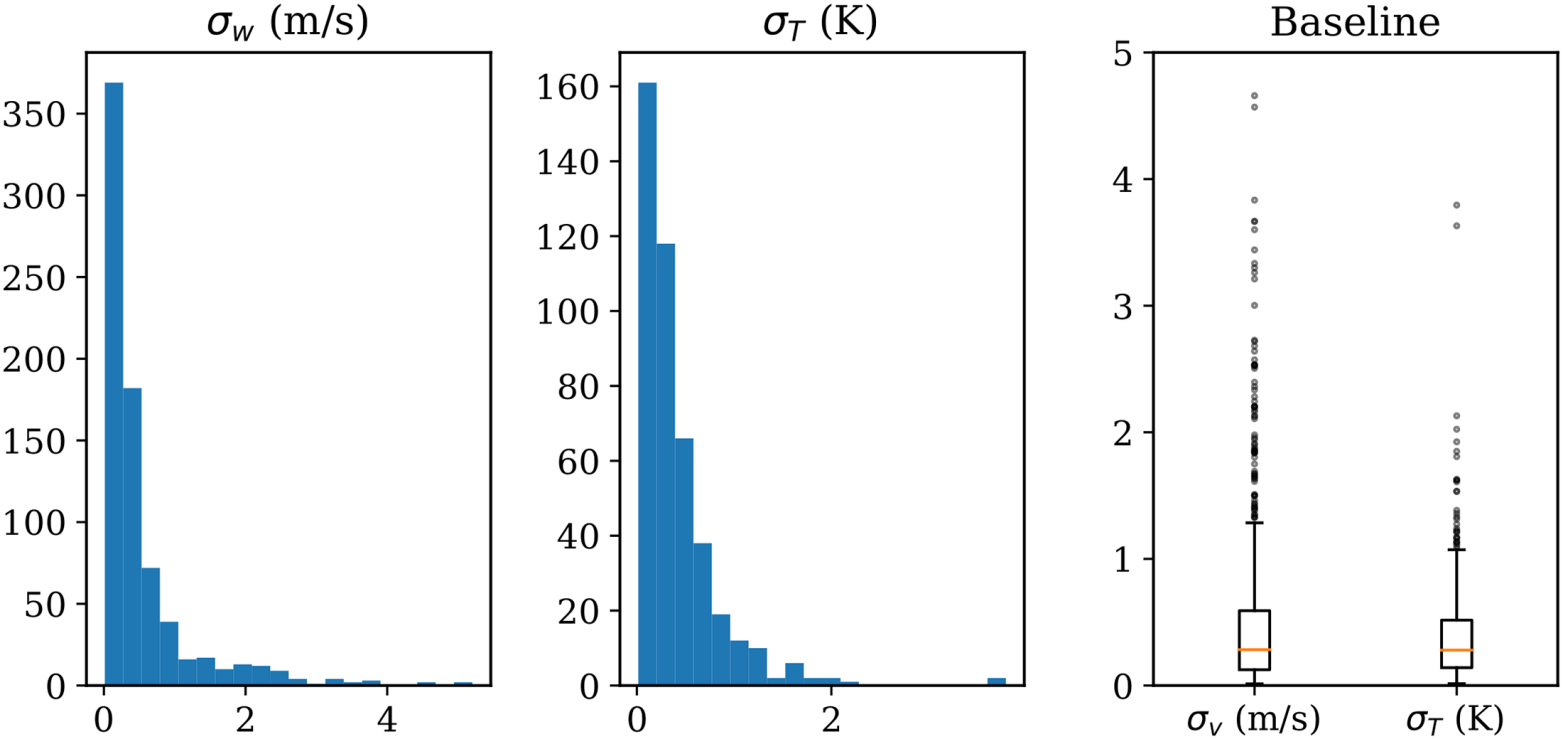
Standard deviation of wind speed and temperature of 100 runs.

In Fig 19, the distribution of standard deviations of wind-grid speed and heading among 100 runs is displayed. Among these runs, the difference is almost negligible: less than 2 m/s for wind speed and 1.5 Kelvin for the temperature.

Examining the resulting fields, the outliers are found to be the points that are far away from the flight paths, where fewer particles would reach. Based on the variations shown in Fig 19, we can conclude that despite the randomness in the model, the MP model always converges to a result with small uncertainties. The results here can be considered as the baseline model for comparison with other studies later in this section.

### 6.2 Data uncertainty

Another important validation is to determine how the quality of observation data affects the wind field estimation. More precisely, it is necessary to ascertain whether the wind grid would be different if some percentage of the observed data is not available. To study this effect, the previous dataset is randomly sampled into several new datasets that contain 80%, 40%, 20%, 10%, and 50% of the total wind observations. Then, the same processes are run to create five different wind fields at 12:00 hours.

**Fig 20:**
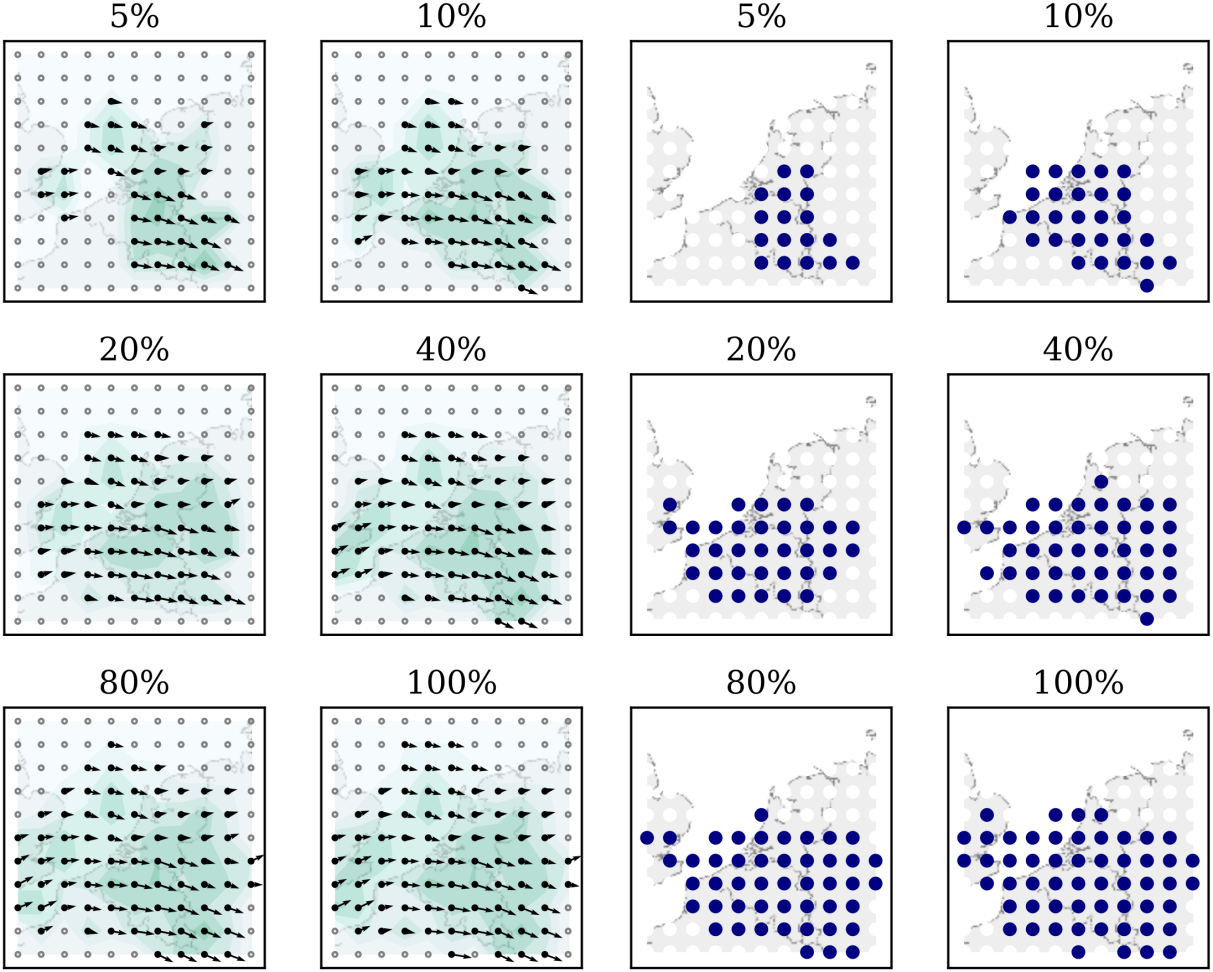
Wind and temperature field at a 8km altitude under different samples.

Fig 20 illustrates the wind and temperature grids, estimated at an altitude of 8km when different percentages of sampled data are used. From the first plot to the last, it is obvious that with increasing observation data samples, the size of the estimated field is increased. At the same time, with increasing sample size, the difference between wind fields is smaller.

**Fig 21:**
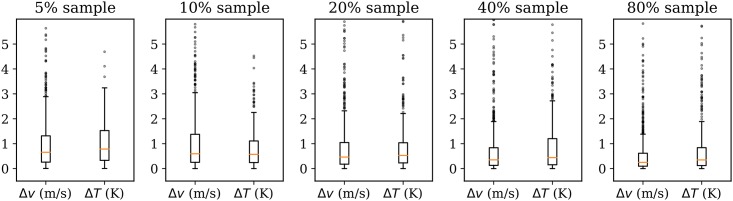
Grid magnitude and heading difference due to sampling.

In order to quantify the differences, the absolute mean differences between wind and temperature are calculated. In Fig 21, the distribution of the entire grid (including all altitude levels) of all wind vectors are compared with the results from the complete data.

Compared to the baseline variance of the model as shown in Fig 19, we can infer that with a loss of up to 90% of the total data (10% sample), the differences are still small. This test indicates that there is an abundant number of observations from aircraft to sustain a stable meteorological field reconstruction. At the same time, even with a large amount of data loss, the MP model can still obtain stable and correct results. By examining the differences of wind and temperature under different sample rates, we can observe that the results of MP model are consistent with the result using the full dataset, even though the size of the fields may be reduced.

### 6.3 Error tolerance

Measurement errors in raw data affect the stability and correctness of the MP model results. We want to quantify the percentage of the errors in data which would produce significant divergence of results. In this experiment, a percentage of the dataset is replaced with random wind vectors that are uniformly distributed between the minimum and maximum wind speeds with headings between 0 and 360 degrees. Temperatures are altered randomly within +/- 20 Kelvin of the originally computed temperature.

**Fig 22:**
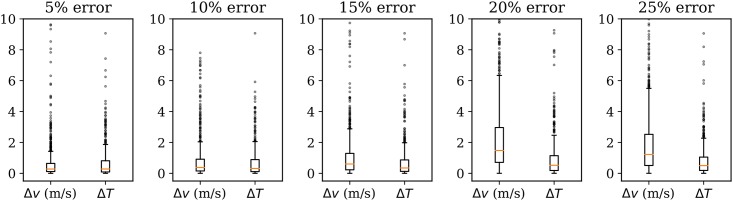
Grid magnitude and heading difference due to error.

In Fig 22, wind grid differences between no assumed error and data error rates of 2%, 4%, 6%, 8%, 10%, and 15% are shown.

**Fig 23:**
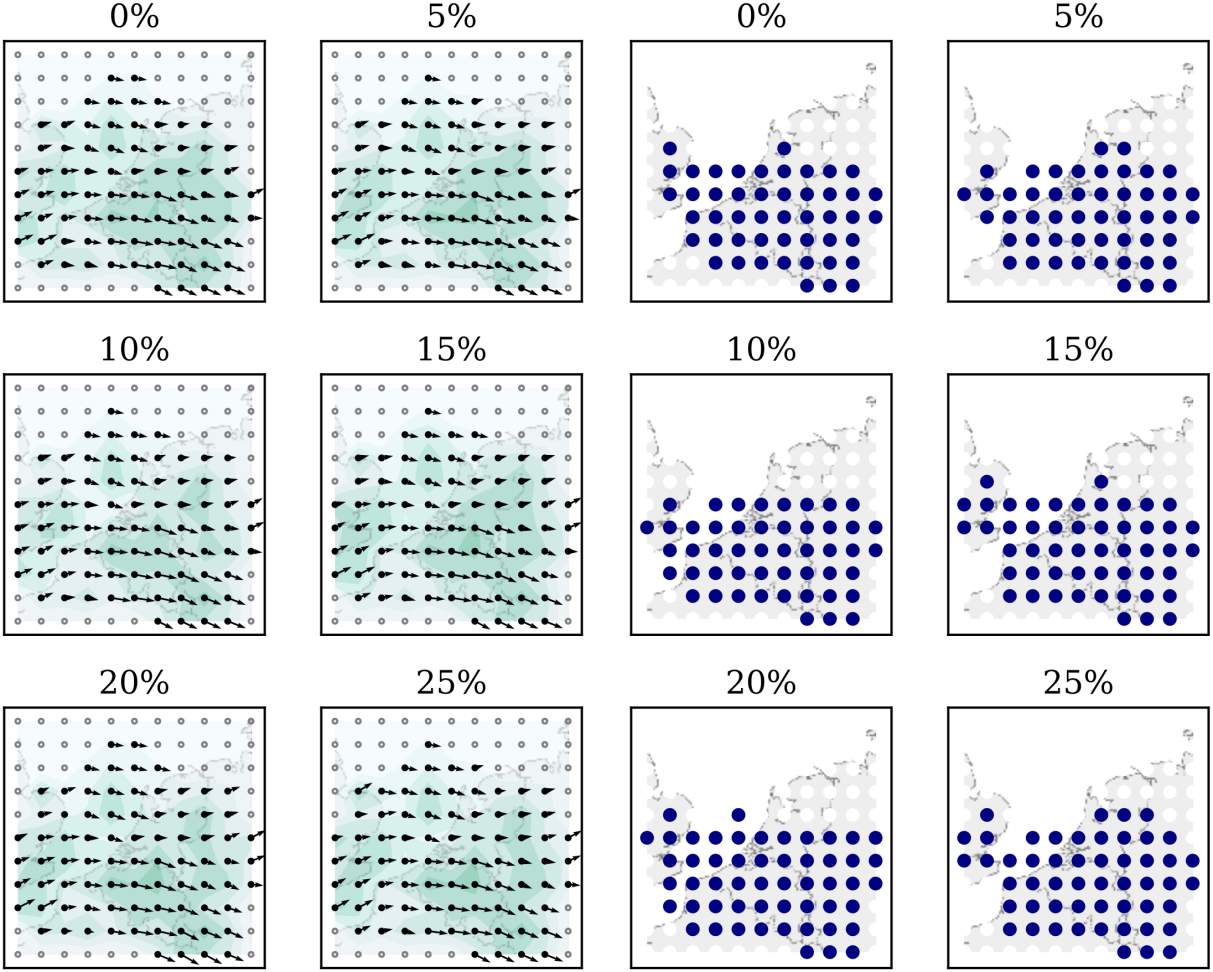
Wind and temperature field at a 8km altitude under different error rate.

With such an aggressive error model, the MP model can maintain a reasonably correct wind field with an error rate of up to approximately 15% (in comparison to the baseline). As a visualization reference, wind and temperature field (8km altitude) under different error rates are shown in Fig 23.

## 7 Summary and discussion

Throughout the paper, we describe the Meteo-Particle model and related methods to construct a wind and temperature field from aircraft position and speed broadcasts. We also propose a Gaussian regression predictor for short-term wind and temperature prediction.

The first contribution of this paper is the method to compute weather information using the aircraft broadcast data. The challenge is not only the decoding of BDS 5,0 and 6,0 messages but rather a complete identification process of the entire Mode-S family of messages. As a third-party observer without the knowledge of Mode-S interrogations, this decoding process can be complex. Previously, we developed the pyModeS decoder to solve this problem, which is used in this paper to obtain accurate airspeed and temperature measurements.

The core of this paper focuses on constructing a model that is able to cope with the chaotic nature of wind, moving aircraft, and non-uniformly distributed observations. The MP model proposed in this paper addresses the stochastic characteristic of wind through particles while maintaining stability through the use of a large number of particles. The MP model uses a stochastic update to propagate wind information to surrounding areas using imaginary particles. With these propagated particles, wind and temperature fields can be estimated in areas where fewer or no measurements are available. Parameters on particle propagation and decay can be tuned in order to control performance. Recommended parameters used in this paper are given in Table 6. They are based on empirical knowledge but not necessarily optimized for all receiver setups.

**Table 6:** Meteo-Particle model parameters used in this paper.

Notation	Parameter	Value	Unit
*σ*_*u*0_	Particle initialization wind variation (u component)	0.2	m/s
*σ*_*v*0_	Particle initialization wind variation (v component)	0.2	m/s
*σ*_*τ*0_	Particle initialization temperature variation	0.1	K
*σ*_*px*_	Particle random walk (x direction)	5	km
*σ*_*py*_	Particle random walk (y direction)	5	km
*σ*_*pz*_	Particle random walk (z direction)	0.1	km
*x*_*b*_	Selection boundary (x direction)	20	km
*y*_*b*_	Selection boundary (y direction)	20	km
*z*_*b*_	Selection boundary (z direction)	500	m
*k*_1_	Measurement acceptance probability factor	3	-
*k*_2_	Particle random walk factor	10	-
*σ*_*α*_	Particle aging parameter	180	s
*σ*_*d*_	Weighting parameter (distance to point of interest)	30	km
*σ*_*d*_	Weighting parameter (distance to origin)	30	km

When reviewing the objectives of the Meteo-Particle model, it can be viewed as a type of data assimilation method. In the introduction of this paper, we mentioned a few existing variational assimilation methods in NWP models, for example, 3DVAR [18] and 4DVAR [19] used by ECMWF. These methods are better suited for large spatiotemporal modeling with data from different observation sources. The MP model focuses on a fast local real-time weather reconstruction, based on aircraft measurements specifically. Without minimizing the cost function as in variational methods, the meteorological grid constructed based on the MP model displays less smoothness than NWP model. For supporting general air traffic studies, this lower level of continuity is not a concern. Sometimes it is not even necessary when only weather along a specific trajectory needs to be constructed.

When comparing the Monte-Carlo based particle approach in MP model to Gaussian weighted interpolation, there are several advantages. The MP model always has the past observation information without the need of storing historical measurement. When constructing wind and temperature a location, we only need to consider a small amount of the particles in adjacent areas, which is faster to compute. Most importantly, the probabilistic observation acceptance mechanism introduced in the MP model ensures the erroneous observations are not used in the system. Thus, they have little influence to the current and future estimations.

In this paper, three assumptions were made before constructing the MP model. The first two assumptions stated that the variation of wind and temperature are small temporally (at the level of hours) and locally (at the level of kilometers). Only under these two conditions, the aggregated states from propagated particles can represent the true weather conditions. Later on, from the example dataset and result produced, we can confirm the validity of these two hypotheses. However, we have to be cautious when applying the model to near-surface scenarios, such as constructing very low altitude wind fields using aircraft data from the takeoff phase. The wind dynamics can be far from locally steady in this situation. In this paper, the lowest altitude was set at 1 km to avoid such conditions, regardless of the actual planetary boundary layer.

The last assumption stated that the error rate in original measurement data is not too large. This is generally guaranteed with the accuracy of pyModeS decoder. Using one-hour ground truth data from a local air traffic controllers, we were able to find out that the error rate of pyModeS on BDS 5,0 and BDS 6,0 message identification is below 0.1%. With this accuracy, only the original aircraft speed measurement error and data transmission error acted as the causes of inaccuracy. However, with the probabilistic error rejection, we showed that the model can handle up to 15% of the artificial error in the data (as shown in Figs 22 and 23). This is well beyond any normal error rate in aircraft surveillance data.

One of the limitations in analysis of the paper is the time period of the data used for validation with GFS data. Ideally, prolonged of periods validation would give a more confident statement. Nevertheless, as illustrated in Figs 16 and 17 and Tables 3 and 4, the randomly chosen dataset already displays a large improvement in terms of accuracy.

## 8 Conclusions

With the increasing accessibility of open aircraft surveillance data from ADS-B and Mode-S, as well as the development of related open-source decoding libraries, exciting new possibilities for research are made available to the research community. In this paper, we proposed a fast, real-time model, the Meteo-Particle (MP) model, to construct real-time wind and temperature fields using aircraft as sensors. Short-term prediction capability is also demonstrated under a Gaussian Process Regression predictor.

At first, using our pyModeS library, raw temperature and wind states are computed from ADS-B and Mode-S down-link data. Then, the MP model can be used to construct wind and temperature fields within the range of the receiver, which is around 300 to 400 kilometers in radius in this paper. The results obtained from MP model is close NWP reanalysis data. For example, when comparing with GFS reanalysis (0.5 degree resolution) data, the absolute mean difference in wind speed, wind direction, and temperature is 2.74 m/s, 6.94°, and 0.64 K respectively. When comparing to ECMWF ERA5 (0.25 degree resolution) data, the differences are 1.99 m/s, 5.37°, and 0.74 K respectively. We also compare the accuracy of inference using MP model and GFS data with unseen data. The mean absolute error of wind speed and temperature estimations are reduced by 67% and 26% when MP model is applied. This increased accuracy indicates the potential benefits for aircraft performance and air traffic management studies.

The Meteo-Particle model demonstrates the validity of using aircraft as large sensor networks to construct a global scale real-time meteorological measurement system for the open research domain. In contrast to the current, proprietary, low update rate AMDAR system, this model and the results proposed in this paper, are fully open to the ATM and the wider scientific community. The implementation of the MP model in Python programming language is shared [28]. Without the need for any new equipment or communication protocols, the implementation of such a system can be enacted using existing technology and data sources. Based on the single receiver demonstrated in this paper, we believe the future research can offer meteorological monitoring capability with a large coverage by using data from existing crowd-sourced receiver networks.
